# Breathing Some New Life into an Old Topic: Chalcogen-Nitrogen π-Heterocycles as Electron Acceptors †

**DOI:** 10.3390/molecules18089850

**Published:** 2013-08-16

**Authors:** Anton V. Lonchakov, Oleg A. Rakitin, Nina P. Gritsan, Andrey V. Zibarev

**Affiliations:** 1 Institute of Chemical Kinetics and Combustion, Russian Academy of Sciences, 630090 Novosibirsk, Russia; E-Mails: lonchakov@kinetics.nsc.ru (A.V.L.); gritsan@kinetics.nsc.ru (N.P.G.); 2 Department of Physics, National Research University—Novosibirsk State University, 630090 Novosibirsk, Russia; 3 Institute of Organic Chemistry, Russian Academy of Sciences, 119991 Moscow, Russia; E-Mail: orakitin@ioc.ac.ru; 4 Institute of Organic Chemistry, Russian Academy of Sciences, 630090 Novosibirsk, Russia

**Keywords:** organic electron acceptors, radical-anion salts, charge-transfer complexes

## Abstract

Recent progress in the design, synthesis and characterization of chalcogen-nitrogen π-heterocycles, mostly 1,2,5-chalcogenadiazoles (chalcogen: S, Se and Te) and their fused derivatives, possessing positive electron affinity is discussed together with their use in preparation of charge-transfer complexes and radical-anion salts—candidate building blocks of molecule-based electrical and magnetic functional materials.

## 1. Introduction

Despite rapid progress in the design, synthesis and characterization of molecule-based functional materials for electronics and spintronics, particularly (photo-) conducting, superconducting and magnetic ones, there is a permanent demand for new building blocks in the field (the relevant literature is too abundant to be cited completely, for selected recent references see [1,2,3,4,5,6,7,8,9,10,11,12,13,14]). Especially interesting possibilities are associated with chalcogen-nitrogen chemistry [15,16,17,18] best known in materials science with polymeric sulfur nitride (SN)_x_ [19]—the first macromolecular metal and low-temperature superconductor [20,21]. Another significant impact of this chemistry in materials science is that a large number of candidate building blocks—the spin and/or charge carriers for molecular functional materials were found amongst chalcogen-nitrogen π-heterocyclic radicals, both neutral and positively charged (*i.e.*, radical cations) [22,23,24,25,26,27,28,29,30,31,32,33,34,35,36,37,38,39,40,41,42,43,44].

The best studied systems are sulfur-nitrogen, or thiazyl, π-heterocyclic radicals and radical cations. Their common structural feature is the SN fragment, *i.e.*, the same moiety that polymeric sulfur nitride (SN)_x_ is composed of. The free radical SN**^·^** (congener of nitric oxide) has never been detected by EPR spectroscopy in its ground ^2^Π_1/2_ state but the EPR spectrum of the thermally accessible excited ^2^Π_3/2_ state has been recorded instead in a gas phase. In condensed phases, this radical seems to be undetectable by EPR spectroscopy due to instability and/or strong g-anisotropy [45,46,47].

The diversity of properties displayed by potential materials based on the open-shell thiazyl species covering magnetic ordering, magnetic bistability and electric conductivity is not observed for any other class of compounds. Particularly, crystalline samples of the neutral thiazyl radicals and their Se congeners revealed spin-canted antiferromagnetism, as well as electrical conductivity in combination with spin-canted antiferromagnetism or ferromagnetism ([48,49,50] and references therein). In the solid state, some π-heterocyclic thiazyl radicals demonstrate spin-state crossover accompanied by magnetic hysteresis. The bistability arises from the coexistence over a temperature range of two-solid state phases, one based on paramagnetic radicals, and the other on their weakly bonded diamagnetic π-dimers. It is believed that magnetically bistable materials can find applications in molecular spintronic devices, *i.e.*, in magneto-thermal switching and information storage devices [30,34,51,52,53].

The Se congeners of π-heterocyclic thiazyl radicals and radical cations are much less studied whereas Te congeners are unknown [22,24,48,49,50,54]. This is due to well-known experimental difficulties associated with (organo)tellurium chemistry (see, for example, [54] and references therein). Conceptually, variation of chalcogens in the chalcogen-nitrogen π-heterocycles does not involve any serious challenge: S, Se and Te substitute each other isostructurally and isoelectronically, and 2,1,3-benzochalcogenadiazoles give representative example [55]. On the other hand, heavier-chalcogen congeners of open-shell thiazyl compounds are of enhanced interest especially in the context of the heavy-atom effect on electrical conductivity and magnetic properties [25,26,27,48,49].

Negatively charged chalcogen-nitrogen π-heterocyclic radicals (*i.e.*, radical anions—RAs), though observed by EPR (S and Se derivatives) in the mid-1960s [18,56,57,58,59,60,61,62,63,64,65], were not used until recently in the design and synthesis of potentional functional materials because of the lack of methods for their isolation. The only related species were RAs of TCNQ-fused 1,2,5-thia(selena)diazoles isolated in the form of salts [66,67,68,69]. However these RAs are normally considered as derivatives of TCNQ, *i.e.,* the well-known precursor of numerous RA salts and charge-transfer (CT) complexes.

**Figure 1:**
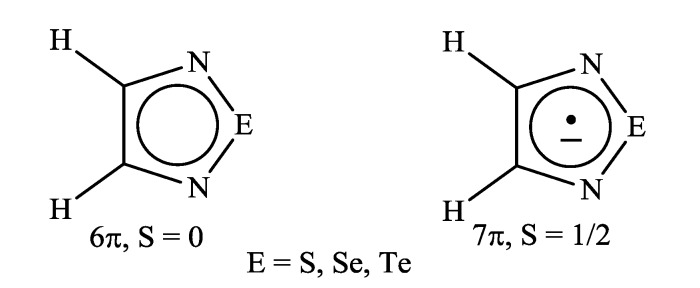
Archetypal 1,2,5-chalcogenadiazoles and their radical anions.

Recently, methods for isolation of genuine chalcogen-nitrogen π-heterocyclic RAs in the form of thermally stable salts have been reported [70,71,72,73,74,75,76,77,78,79,80]. Structurally, the RAs belonged mostly to the 1,2,5-chalcogenadiazole ring system (Figure 1) but not only. An important feature of 1,2,5-chalcogenadiazoles and many other related π-heterocycles is their positive electron affinity. As a consequence, RAs are more thermodynamically stable than neutral precursors. The RAs reveal no propensity to isomerization or monomolecular decay. These features provide a basis for their isolation in the form of salts despite the obvious kinetic activity of these species, particularly towards atmospheric moisture and oxygen.

The aforementioned 1,2,5-chalcogenadiazolidyl salts are the first representatives of a novel class of paramagnetic chemical compounds. Potentially, this class is broad since derivatives of some other chalcogen-nitrogen π-heterocyclic systems can be used as precursors of stable RAs. Due to this, a distinctive feature of this class can be specified as chalcogen-nitrogen π-heterocyclic anion bearing unpaired π-electron, *i.e.*, possessing a spin S = 1/2. No restrictions are imposed on the cation.

Persistent RAs of acyclic analogues of 1,2,5-thiadiazoles, *i.e.*, sulfur diimides R-N=S=N-R (R = Alk, Ar), and their complexes with metal (Cr, Mo, W) carbonyls have also been known for a long time from solution EPR experiments [81] but have not been isolated to date.

In this review, we outline recent progress in the design, synthesis and characterization of chalcogen-nitrogen π-heterocycles, mostly 1,2,5-chalcogenadiazoles (chalcogen: S, Se and Te), and their fused derivatives, possessing positive electron affinity and discuss their use in the preparation of RA salts and CT complexes as candidate building blocks for molecule-based magnetic and conducting functional materials. The review deals first of all with the fundamental chemistry and physics of the heterocycles, and not with materials science. All possible applications mentioned in the text await realization of their potential, and in this aspect the field is in its infancy.

In some important aspects, this new field conceptually overlaps with, and continues development of, the chemistry of polysulfur-nitrogen heterocyclic compounds, *i.e.*, compounds with unusually high proportions of sulfur and nitrogen atoms with respect to carbon atoms, originated by Charles W. Rees in the early 1980s [82,83,84,85,86].

## 2. Electron Affinity and Redox Properties

### 2.1. Electron Affinity

First electron affinity (EA) [87] and first ionization energy (IE) are fundamental physical properties of molecules which are very important not only for their chemical reactivity but also for many of their applications in the field of functional materials. According to the Koopmans’ theorem, first vertical EA and IE are numerically equal to the energies of the LUMO and HOMO (the frontier MOs) taken with the opposite signs, respectively. Basic research of dependence of both EA and IE on molecular composition and structure is still of significant scientific interest. Whereas IE can be only positive, EA can be both positive and negative. Especially interesting is rather rare positive EA.

According to quantum chemical calculations, the EA of many chalcogen-nitrogen π-heterocycles, both known and unknown, is positive, *i.e.*, RAs are thermodynamically more stable than neutral precursors, with the exact numerical results depending on the level of theory [88]. Test calculations of gas-phase adiabatic EA of selected chalcogen-nitrogen π-heterocycles and TCNE at a number of different levels of the theory revealed that resource-economic (U)B3LYP/6-31+G(d) approach performs reasonably well even in comparison with the G3B3 one which accurately reproduced the experimental EA of TCNE. Correction for ZPE hardly affects the results (Table 1) [88,89]. Due to this, compounds of interest, mostly derivatives of 1,2,5-thia- and selenadiazoles presented below, were calculated at the (U)B3LYP/6-31+G(d) level of theory without ZPE correction. For generality, calculations also covered simple 1,2,5-telluradiazoles and some other chalcogen-nitrogen π-heterocycles which do not belong to 1,2,5-chalcogenadiazole ring system. Particularly, ring systems isomeric to 1,2,5-chalcogenadiazole one (chalcogen: S, Se) were taken into account as well as [1,8-c,d][1,2,6]naphthothiadiazine (known to form stable RA [63,65]) and its derivatives (Table 2, Table 3, Table 4, Table 5 and Table 6) [88].

**Table 1:** Adiabatic EA (eV) of referent compounds calculated with/without ZPE correction [89].

Method/Compound	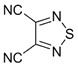	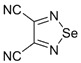	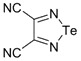	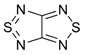	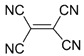
MP2/6-311+G(3df,2p) *^a^*	0.94	1.13	1.25 *^b^*	1.16	2.38
PMP2/6-311+G(3df,2p) *^a^*	1.33	1.54	1.67 *^b^*	1.48	2.93
(U)B3LYP/6-31+G(d)	1.91/1.84	2.01/1.94	2.16/2.10 *^c^*	2.19/2.14	3.51/3.48
(U)B3LYP/6-311+G(3df,2p) *^a^*	1.86	2.04	2.05 *^b^*	2.07	3.50
G3B3	1.74	( *^d^*)	( *^d^*)	1.96	3.17 *^e^*

*^a^* Geometry optimization of neutral molecule and its RA at the BP86 level. *^b^* Basis for Te: def2-TZVPPD with ECP. *^c^* Basis for Te: def2-SVPD with ECP. *^d^* For Se and Te derivatives, G3B3 calculations are impossible because a number of necessary parameters are unavailable. *^e^* For TCNE, experimental gas-phase value is 3.17 eV [90].

The archetypal 1,2,5- and 1,2,3-thiadiazoles and their Se congeners possess small positive EA and strong electron-withdrawing substituents, e.g., CN are necessary to enlarge it to *ca.* 2 eV. On the contrary, 1,2,4- and 1,3,4-thiadiazoles and the Se analogue of the latter have negative EA. Electron-withdrawing substituents CF_3_ and CN change the sign of EA of these compounds and significantly enlarge its value. With the same substituents R (R = CF_3_, CN), derivatives of 1,2,3-, 1,2,4- and 1,3,4-chalcogenadiazole systems are better electron acceptors than those of 1,2,5-chalcogenadiazole system (chalcogen: S, Se), and Se and Te derivatives are better acceptors than S ones (Table 2). Overall, the nature of the chalcogen, isomerism and substitution pattern are important in determining their EA. According to the calculations, an interesting peculiarity is associated with 1,2,3-selenadiazole where on formation of the RA the Se-N bond spontaneously dissociates.

**Table 2:** (U)B3LYP/6-31+G(d) adiabatic EA (eV) of the archetypal 1,2,3-, 1,2,4-, 1,2,5- and 1,3,4-thiadiazoles, their Se and Te congeners and selected derivatives [72,88]. *^a^*

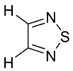	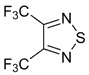	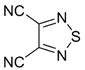	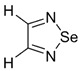	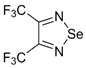	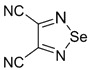
0.05	1.32	1.84	0.22	1.42	1.94
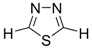	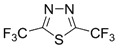	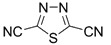	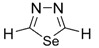	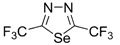	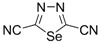
−0.35	1.47	2.20	−0.15	1.55	2.25
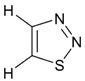	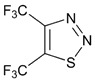	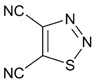	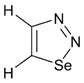	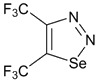	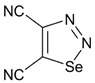
0.08	1.63	2.23	( *^b^*)	1.74	2.31
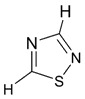	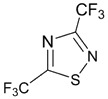	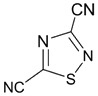	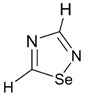	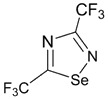	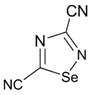
−0.13	1.44	2.05	0.01	1.54	2.11
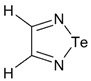	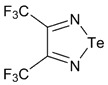	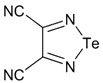			
0.75	1.58	2.10			

*^a^* Basis for Te: def2-SVP with ECP. *^b^* The Se-N bond spontaneously dissociates in the RA.

Benzo-fused derivatives of 1,2,5-chalcogenadiazoles (*i.e.*, 2,1,3-benzochalcogenadiazoles) reveal EAs enlarged by *ca.* 0.9 eV as compared with their monocyclic prototypes. Electron-withdrawing F, NO_2_ and CF_3_ substituents in the carbocycles enlarge the discussed property further (Table 3).

EA of 2,1,3-benzothiadiazoles can also be sufficiently enlarged by their conversion into 1-oxides (Table 3) [88].

**Table 3:** (U)B3LYP/6-31+G(d) adiabatic EA (eV) of 2,1,3-benzochalcogenadiazoles and their selected derivatives [56,72,88]. *^a^*

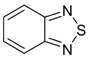	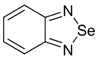		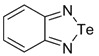	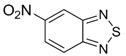
0.95	1.06		1.15	2.11
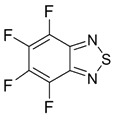	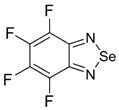		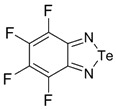	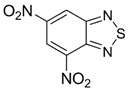
1.57	1.67		1.83	2.86
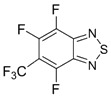	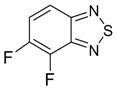		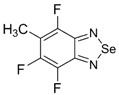	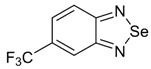
1.91	1.24		1.42	1.50
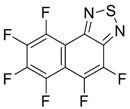		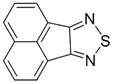		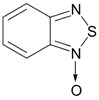
1.67		0.95		1.29

*^a^* Basis for Te: def2-SVP with ECP.

Another way to enlarge EA of benzo- and naphtho-fused 1,2,5-thiadiazoles, as well as EA of related [1,8-c,d][1,2,6]naphthothiadiazines, by *ca.* 0.8–0.9 eV is substitution of their carbon atoms by nitrogen ones. In the naphthothiadiazine series, it allows to achieve EA of 3 eV (Table 4). The corresponding derivative is unknown thus being interesting synthetic challenge.

**Table 4:** (U)B3LYP/6-31+G(d) adiabatic EA (eV) of 1,2,5-thiadiazoles and 1,2,6-thiadiazines fused with selected aza-benzenes and -naphthalenes [72,88].

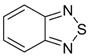	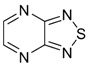	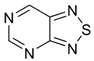	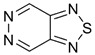	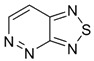
0.95	1.75	1.75	1.79	1.88
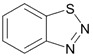	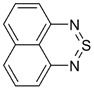	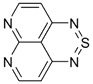	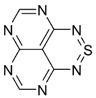	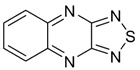
0.58	1.77	2.45	2.99	2.21

Compounds containing two 1,2,5-thiadiazole rings, or this ring in combination with its Se or O analogues, fused directly or via benzene or aza-benzene cycles, also possess high positive EA. In the case of known bis([1,2,5]thiadiazolo)[3,4-b;3’,4’-e]pyrazine [91] and its unknown O congener, calculated EA values exceeded 3 eV being markedly bigger for the later (Table 5). Another promising compound with EA > 2 eV is 1,3,5,7-tetrathia-2,4,6,8-tetraza-2a-azulene described in literature [83].

**Table 5:** (U)B3LYP/6-31+G(d) adiabatic EA (eV) of compounds with two or three 1,2,5-thiadiazole rings, their selected O and Se congeners and 1,3,5,7-tetrathia-2,4,6,8-tetraza-2a-azulene [72,73,88].

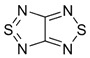	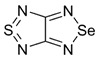	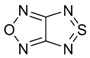	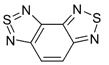	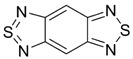
2.14	2.19	2.44	1.24	2.26
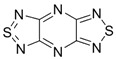	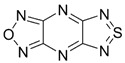	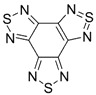	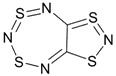	
3.04	3.39	1.24	2.32	

The highest EA values were however obtained not for 1,2,5-chalcogenadiazoles but for derivatives of (6*H*-1,2,3-benzodithiazol-6-ylidene)malononitrile (Table 6). In known archetypal compound [84], substitution of carbon atoms by nitrogen ones should lead to EA as high as 3.46 eV, *i.e.*, to the value obtained at the same level of theory for TCNE (Table 1). These derivatives are unknown thus representing one more interesting synthetic challenge in the field.

**Table 6:** (U)B3LYP/6-31+G(d) adiabatic EA (eV) of (6*H*-1,2,3-benzodithiazol-6-ylidene)malononitrile and its selected derivatives [80,88].

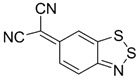	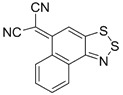	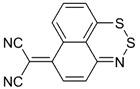	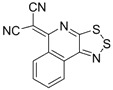
2.69	2.55	2.85	2.66
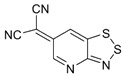	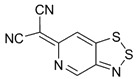	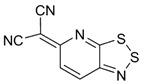	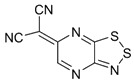
2.94	2.94	2.82	3.07
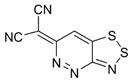	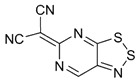	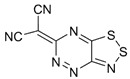	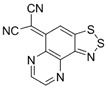
3.31	3.10	3.46	2.69
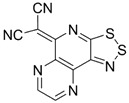	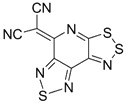	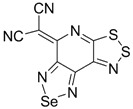	
2.81	2.88	2.81	

Overall, one can conclude that various chalcogen-nitrogen π-heterocycles are promising precursors of thermodynamically stable RAs. In many cases studied at the (U)B3LYP/6-31+G(d) level of theory, their adiabatic EA exceeded 2 eV and for discussed derivatives of (6*H*-1,2,3-benzodithiazol-6-ylidene)malononitrile even 3 eV.

An interesting trend is that in the isostructural series of 1,2,5-chalcogenadiazoles and their benzo-fused derivatives (chalcogen: S, Se, Te) the molecular EA increases with atomic number of the chalcogen, *i.e.*, from S to Te despite the fact that atomic EA and Allen electronegativity decreases in this sequence as 2.08 (S), 2.02 (Se) and 1.97 (Te) eV, and 2.59 (S), 2.42 (Se) and 2.16 (Te), respectively. As follows from Table 1, this result is not an artifact of the DFT approach.

Within the structural classes, the π-MOs of the discussed heterocycles are isolobal, *i.e.*, their shapes are invariant to the nature of chalcogen atoms [73].

Overall, according to the calculations, broad structural variation of the archetypal chalcogen-nitrogen π-heterocycles, including substitution, achievable with current synthetic methods should allow control of the EA of compounds of interest.

### 2.2. Redox Properties

Under electrochemical conditions, 1,2,5-chalcogenadiazoles and some other related chalcogen-nitrogen π-heterocycles readily form persistent RAs (Table 7), in most cases characterized by EPR in combination with quantum chemical calculations. The most well studied are sulfur-nitrogen derivatives [18,56,70,92,93,94,95,96].

**Table 7:** Electrochemical potential -1/0 (V) (E_p_ vs SCE) of selected chalcogen-nitrogen π-heterocycles. *^a^*

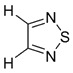	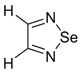	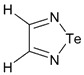	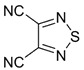	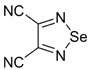
−2.17	(*^b^*)	(*^b^*)	−1.07	−1.03
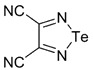	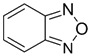	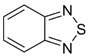	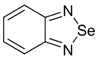	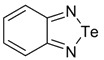
(*^c^*)	−1.48	(−1.51)–(−1.53) *^d^*	(−1.30)–(−1.38) *^d^*	(*^b^*)
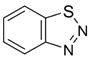	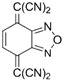	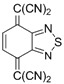	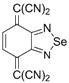	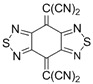
−1.64	0.22	0.12	0.04	−0.02
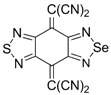	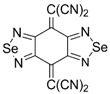	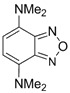	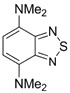	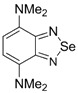
−0.12	−0.23	−1.73	−1.72	−1.57
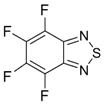	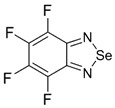	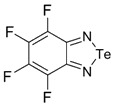	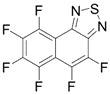	
−1.21	−1.08	(*^b^*)	−1.29	
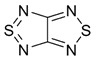	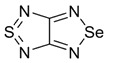	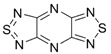	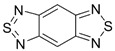	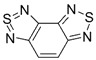
−0.59	−0.53	0.10	(*^b^*)	−1.45
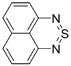	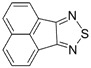	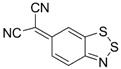	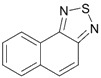	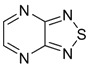
−0.96	−1.71	−0.46	−1.64	−0.87
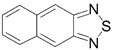	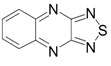	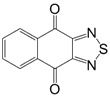	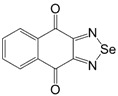	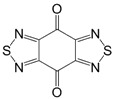
−1.16	−0.46	−0.70, *^e^* −0.80 *^f^*	−0.80 *^e^*	−0.58 *^e^*
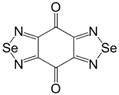	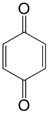	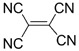	TCNQ	
−0.67*^e^*	−0.43	0.15	0.18	

*^a^* All potentials are given for MeCN solutions and *vs.* SCE (E_p_ *vs.* SCE) unless otherwise indicated, the data are taken from [18,56,71,77,80,94,95,96]. *^b^* Unknown. *^c^* Cyclic voltammetry experiments on this telluradiazole did not give definitive results. From the data for S and Se congeners, its -1/0 electrochemical potential in MeCN can be estimated as ∼−1.0 V. The cyclic voltammogram of the compound had complex form which depends strongly upon solvent (MeCN or DMF) and potential sweep rate, and observed -1/0 peak (at −0.94 and −0.78 V in MeCN and DMF, respectively) seemed to be irreversible [97]. *^d^* From different measurements. *^e^* Potentials are given for DMF solutions [98]. *^f^* Potential is given for CH_2_Cl_2_ solution [99].

As with the EA, it is seen (Table 7) that 1) the −1/0 electrochemical potential of the heterocycles varies in a broad range depending on their structure and composition; and 2) in the isostructural series of 2,1,3-benzooxa- and -chalcogenadiazoles (chalcogen: S, Se) electron-acceptor ability grows with the atomic number of X (X = O, S, Se).

One of the most interesting findings is however stability of RAs of polyfluorinated 2,1,3-benzothia(selena)diazoles and their derivatives [56]. In contrast to RAs of polyfluorinated (hetero)aromatics such as pentafluoropyridine and octafluoronaphthalene which are highly unstable in solution at ambient temperature and cannot be detected by conventional EPR, these RAs are long-lived under electrochemical conditions in MeCN, and especially in DMF, at 295 K [56]. This motivates further work towards their isolation in the form of stable salts.

## 3. Synthesis

For 1,2,5-chalcogenadiazoles (chalcogen: S, Se, Te), only recent progress in synthesis is discussed below. Since target RAs can be additionally stabilized by coordination to metal centers (*cf.* [57,58]), the coordination ability of 1,2,5-chalcogenadiazoles is also highlighted in some cases.

As follows from Table 2, Table 3, Table 4, Table 5 and Table 6, some other chalcogen-nitrogen π-heterocycles are expected to be precursors of stable RAs. Amongst them, 1,2,6-thia(selena)diazines (known to form stable RAs [63,65]) belong to the most promising and are already involved in ongoing research by the authors. For this reason, current progress in their synthesis is also briefly discussed.

### 3.1. 1,2,5-Thiadiazoles

**Scheme 1:**
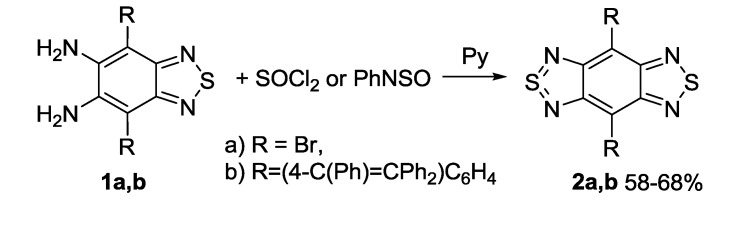
Synthesis of 1,2,5-thiadiazoles **2** from *ortho*-diamino derivatives **1**.

The methods for synthesis of 1,2,5-thiadiazoles and their benzo-fused derivatives (2,1,3-benzothiadiazoles) are well-known [100,101,102,103]. The formation of a 1,2,5-thiadiazole ring from compounds containing two amino groups in *ortho*-positions is the most common way. Thionyl chloride [104] and its derivative *N*-sulfinylaniline allowing milder reaction conditions [105] are normally used in this reaction (Scheme 1).

**Scheme 2:**
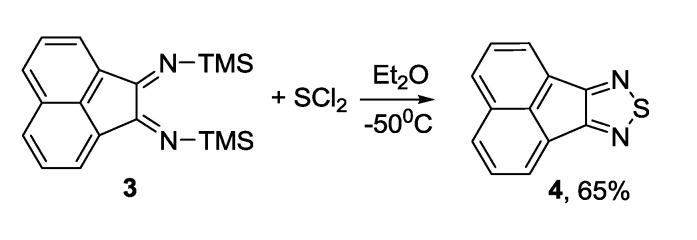
Synthesis of acenaphtho[1,2-c]thiadiazole **4**.

To obtain acenaphtho[1,2-c]thiadiazole **4**, 1,2-bis(trimethylsilyl)iminoacenaphthene **3** was reacted with sulfur dichloride, the isolated yield was 65% [106] (Scheme 2).

**Scheme 3:**
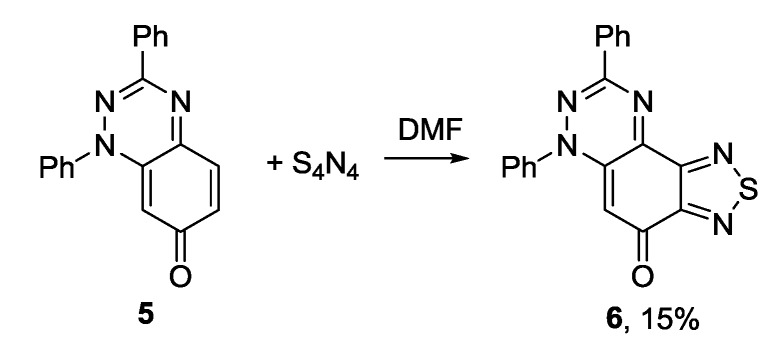
Synthesis of 1,2,5-thiadiazole **6** from triazinone **5** and S_4_N_4_.

1,2,5-Thiadiazole ring can be fused with triazinone **5** using tetrasulfur tetranitride as NSN transfer reagent to produce [5,6-c]thiadiazolo-7-oxo-1,3-diphenyl-1,2,4-benzotriazine **6** however in low yield [107] (Scheme 3).

**Figure 2:**
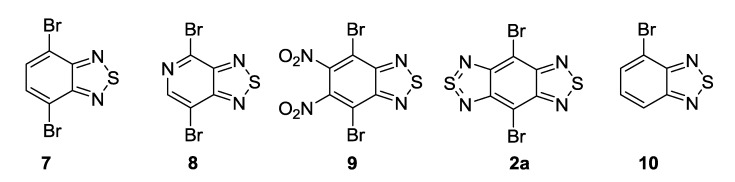
2,1,3-Benzo(pyrido)thiadiazoles involved in cross-coupling functionalization.

The recent synthesis of new benzothiadiazoles containing π-extended photoluminiscent substituents was based on the reactions of commercial 4,7-dibromo-2,1,3-benzothiadiazole **7** and related compounds **2b** and **8**–**10** (Figure 2). The chemistry of compounds **2b**, **7**–**10** was extensively developed (for review, see [103]). The most efforts have been focused on the inclusion of aromatic, heteroaromatic and alkyne substituents into positions 4 and 7 of the benzene or pyridine rings by Suzuki, Stille and Sonogashira cross-couplings.

**Scheme 4:**
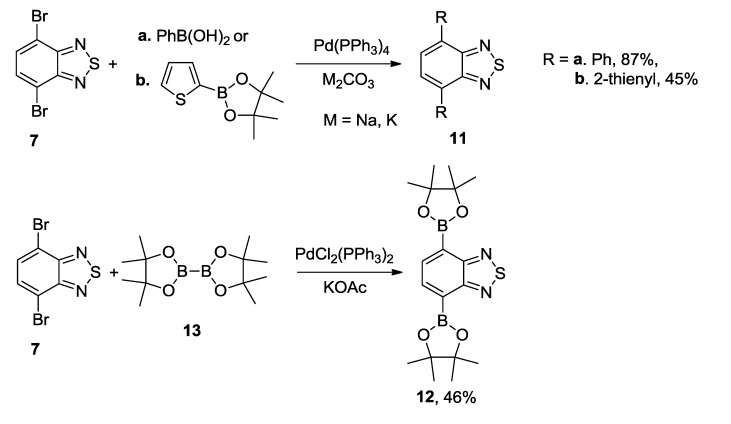
Reaction of 4,7-dibromo-2,1,3-benzothiadiazole **7** with arylboronic acids and their esters.

The Suzuki cross-coupling of compound **7** with arylboronic acids or their cyclic esters in the presence of Pd(PPh_3_)_4_ and alkali metal carbonates led to bis-adducts in moderate to high yields (Scheme 4) [108,109]. Compound **12** with two activated boronic ester groups has been prepared from **7** and bis(pinacolato)diboron **13** in the presence of PdCl_2_(PPh_3_)_2_ and potassium acetate in dioxane, and further used in the synthesis of conjugated polymers for high-performance ambipolar organic thin-film transistors [110,111].

**Scheme 5:**
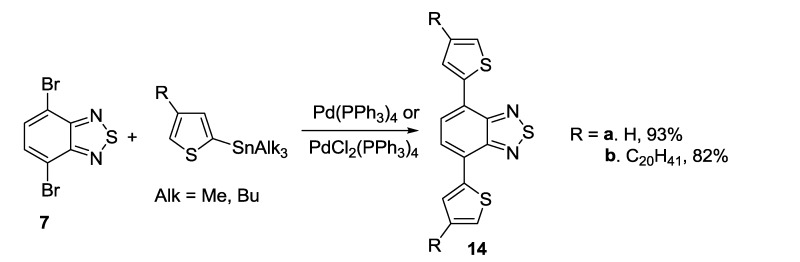
Bis-hetarylation of 4,7-dibromo-2,1,3-benzothiadiazole **7** with trialkylstannyl-thiophenes.

The Stille coupling of compound **7** with thienyl trialkylstannanes in the presence of PdCl_2_(PPh_3_)_2_ [112] or Pd(PPh_3_)_4_ [113] gave bis(thienyl)benzothiadiazoles **14** in high yields (Scheme 5).

**Scheme 6:**
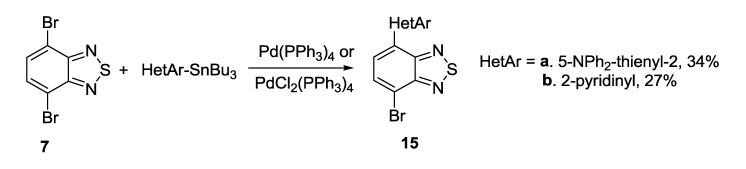
Mono-hetarylation of 4,7-dibromo-2,1,3-benzothiadiazole **7** with hetarylstannanes.

Mono-thienyl- [114] or pyridinyl- [115] substituted benzothiadiazoles **15** were prepared by the same method although the yields were much lower (Scheme 6).

**Scheme 7:**
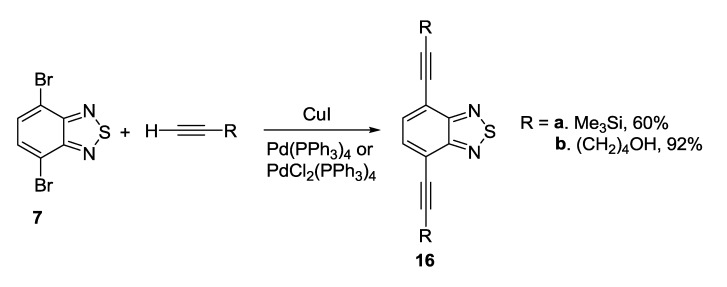
Synthesis of alkynyl substituted 2,1,3-benzothiadiazoles **16**.

The alkynyl units were attached to benzothiadiazole ring by using the Sonogashira coupling of dibromo derivative **7** with alkynes and palladium catalyst [116,117] (Scheme 7).

**Scheme 8:**
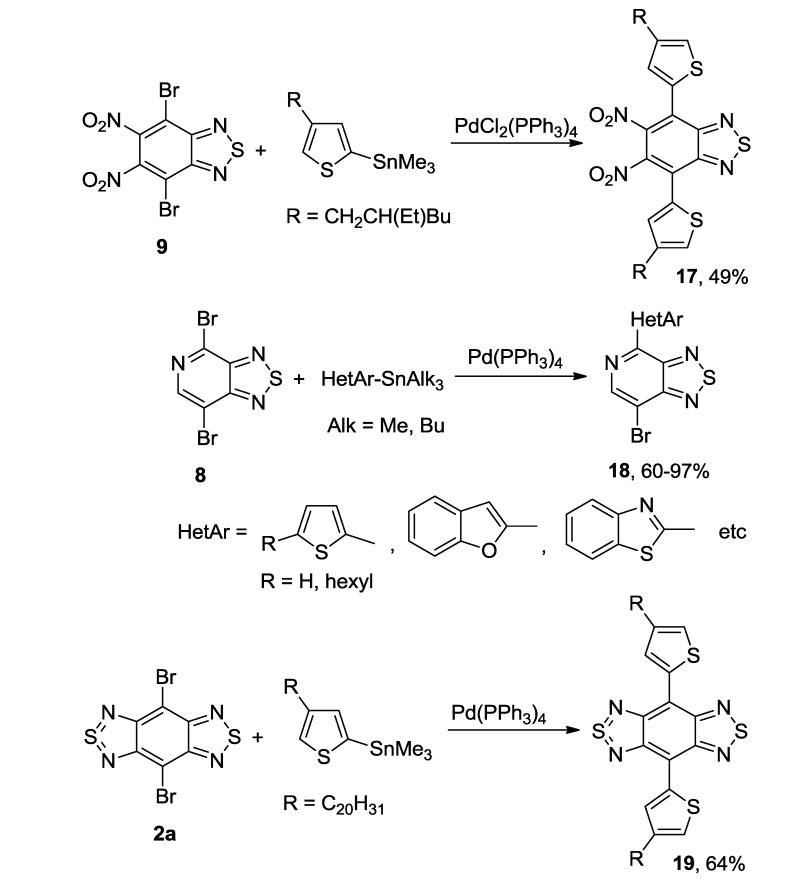
Synthesis of thienyl-substituted 2,1,3-benzo(pyrido)thiadiazoles and related compounds **17**–**19** by the Stille coupling.

The Stille coupling was the most frequently used procedure for preparation of mono- and bis-thienyl-substituted thiadiazoles from a number of dibrominated precursors and trialkylstannanes [118,119,120,121]; in the case of pyridothiadiazole **8** only one bromine atom was substituted (Scheme 8).

**Scheme 9:**
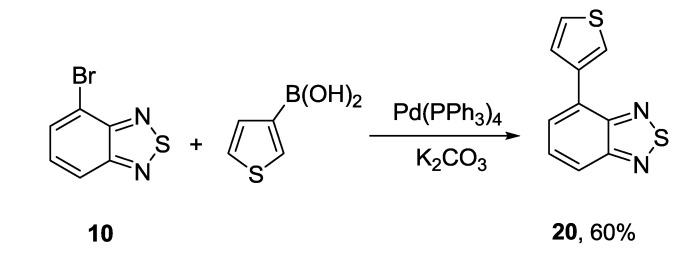
Reaction of 4-bromo-2,1,3-benzothiadiazole **10** with 3-thienylboronic acid.

The Suzuki coupling was found to be useful approach for the synthesis of thienyl derivative **20** from 4-bromo-2,1,3-benzothiadiazole **10**, in that case rarely employed 3-thienylboronic acid was involved in the reaction [122] (Scheme 9).

**Scheme 10:**
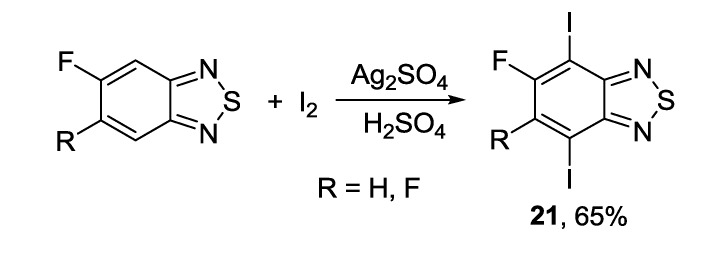
Iodination of fluorinated 2,1,3-benzothiadiazoles.

To facilitate the Stille coupling reaction, a new approach for iodination of mono- and di-fluoro substituted benzothiadiazoles **21** was proposed [123]. The synthesis was performed successfully by a single-step Barker-Waters procedure in the presence of AgSO_4_ and iodine in concentrated sulfuric acid at high temperature, and the target products were isolated in 65% yields (Scheme 10).

**Scheme 11:**
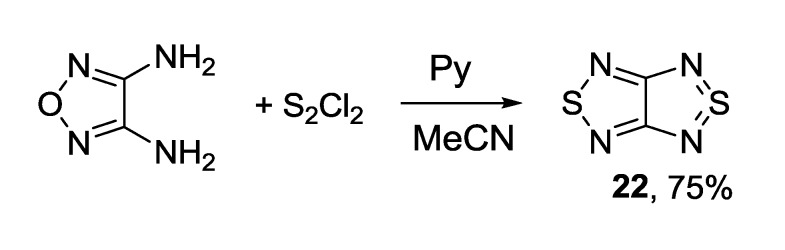
Synthesis of [1,2,5]thiadiazolo[3,4-*c*][1,2,5]thiadiazole **22** from 3,4-diamino-1,2,5-oxadiazole.

Interaction between 3,4-diamino-1,2,5-oxadiazole and sulfur monochloride in pyridine gave [1,2,5]thiadiazolo[3,4-*c*][1,2,5]thiadiazole **22** in high yield (Scheme 11) as the first example of direct exchange of oxygen atom with a sulfur atom in a 1,2,5-oxadiazole ring [89].

**Scheme 12:**
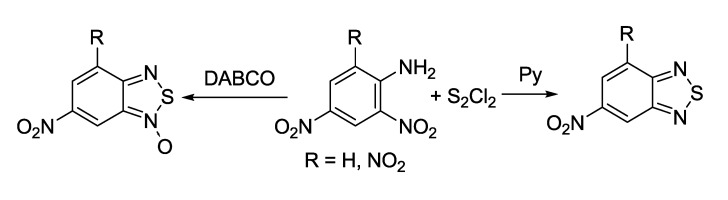
Synthesis of benzo-fused 1,2,5-thiadiazoles and their 2-oxides from *ortho*-nitroanilines.

Reaction of vicinal nitroamines with sulfur monochloride in the presence of organic bases provides a short and convenient synthetic approach to fused 1,2,5-thiadiazoles and their 2-oxides (Scheme 12) [124].

2,1,3-Benzothiadiazole forms molecular complex with trimeric perfluoro-*ortho*-phenylenemercury (anticrown compound) [125]; its 4-OH and -NH_2_ derivatives were used as ligands in a number of iridium complexes [106].

### 3.2. 1,2,5-Selenadiazoles

**Scheme 13:**
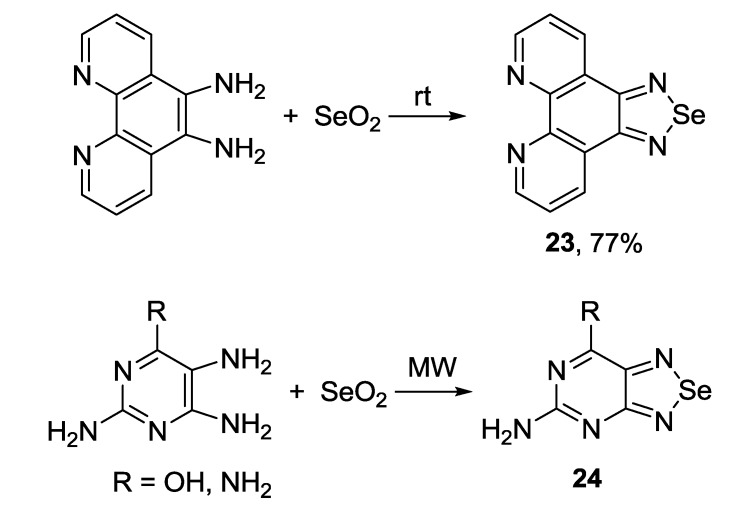
Synthesis of fused 1,2,5-selenadiazoles with selenium dioxide.

*ortho*-Diamines are the most suitable precursors for the preparation of fused 1,2,5-selenadiazoles by reaction with selenium dioxide. Solid-phase synthesis of 1,10-phenanthrolinoselediazole **23** at room temperature [126] and microwave irradiation-assisted preparation of [1,2,5]selenadiazolo[3,4-*d*]pyrimidine **24** [127,128] have been reported (Scheme 13).

**Scheme 14:**
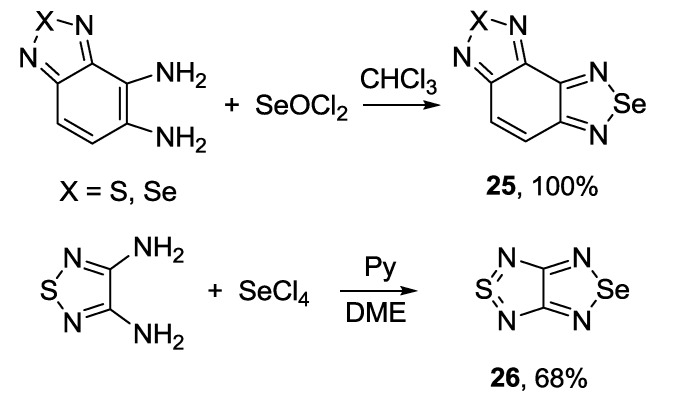
Synthesis of fused 1,2,5-selenadiazoles with selenium oxychloride and selenium tetrachloride.

Selenium oxychloride [129] and selenium tetrachloride [77] were also used for the synthesis of 1,2,5-selenadiazoles fused with other chalcogen-nitrogen heterocycles (chalcogen: S, Se), although rarely (Scheme 14).

**Scheme 15:**
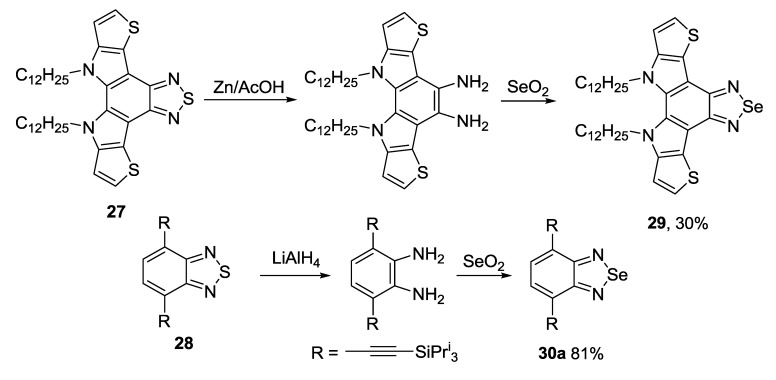
Synthesis of fused 1,2,5-selenadiazoles from 1,2,5-thiadiazoles.

The 1,2,5-chalcogenadiazole ring (chalcogen: S, Se) is a well-known protecting group for *ortho*-diamines. In some cases *ortho*-diamines prepared from fused 1,2,5-thiadiazoles were involved in the reaction with selenium dioxide thus allowing two-step conversion of 1,2,5-thiadiazoles **27**,**28** into their Se congeners **29**,**30** [130,131]. Reducing agents, as well as yields of target 1,2,5-selenadiazoles, may vary significantly (Scheme 15).

**Scheme 16:**
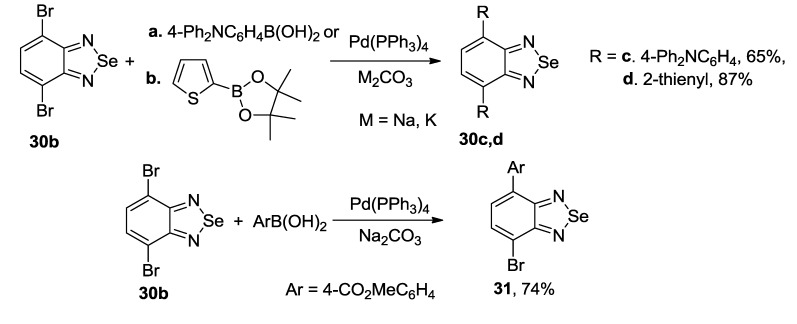
Reaction of 4,7-dibromo-2,1,3-benzoselenadiazole **30b** with arylboronic acids and their esters.

The most common precursor for the synthesis of 4,7-disubstituted 2,1,3-benzoselenadiazoles is 4,7-dibromo derivative **30b**. As for 2,1,3-benzothiadiazoles, the Suzuki and Stille couplings were the most frequently employed procedures. Arylboronic acid [132] and 2-(4-hexylthiophen-2-yl)-4,4,5,5-tetramethyl-1,3,2-dioxaborolane [133] were successfully used in the Suzuki coupling with **30b** to give corresponding benzoselenadiazoles **30c,d** in moderate to high yields (Scheme 16). In some cases mono-adducts **31** may be isolated [134].

A number of 2,1,3-benzoselenadiazole-containing copolymers was prepared from compound **30b** and dioxaborolanes using Pd(PPh_3_)_4_ and K_2_CO_3_ as a catalysts in toluene by heating or under microwave irradiation [135,136].

**Scheme 17:**
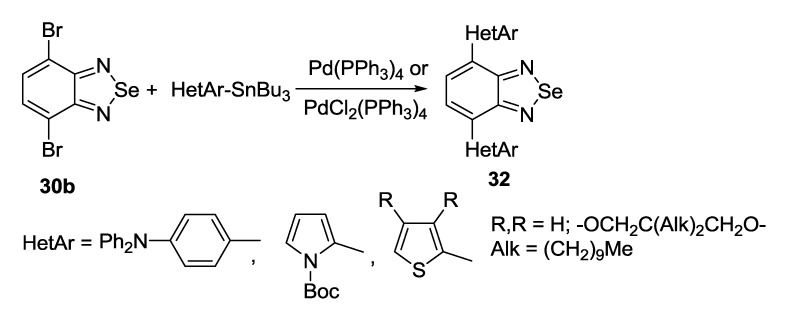
Synthesis of (het)aryl substituted 2,1,3-benzoselenadiazoles **32**.

The Stille coupling is using more frequently for the synthesis of substituted 2,1,3-benzoselenadiazoles than the Suzuki coupling. 4,7-Bis(aryl)- [137], bis(pyrrolyl)- [138] and bis(thienyl)- [139,140,141] 2,1,3-benzoselenadiazoles **32** were successfully prepared by treatment of dibromide **30b** with corresponding tributylstannanes and palladium catalysts (Scheme 17).

Also, copolymers containing thiophene and benzoselenadiazole moieties were synthesized by the Stille coupling of **30b** and trimethylstannanes in the presence of Pd_2_(dba)_3_ and P(*o*-tolyl)_3_ [142,143].

**Figure 3:**
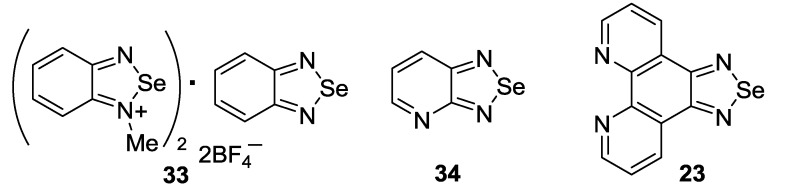
Selenadiazole derivatives **23**, **33** and **34**.

Benzoselenadiazole-containing complexes and self-assembled systems revealed supramolecular structures with unusual and important properties. Particularly, salt **33** [144] and complex of 2,1,3-benzoselenadiazole with HgCl_2_ [145], as well as Ag(I), Cu(I), Cu(II) and Co(II) complexes of 1,2,5-selenadiazolopyridine **34** [146] and ruthenium polypyridine complex of 1,10-phenanthrolinoselenadiazole **23** [147] (Figure 3), were described.

### 3.3. 1,2,5-Telluradiazoles

It should be emphasized that the synthetic chemistry of 1,2,5-telluradiazoles is much more difficult experimentally compared with the chemistry of their Se and S congeners ([97,148] and references therein).

**Scheme 18:**
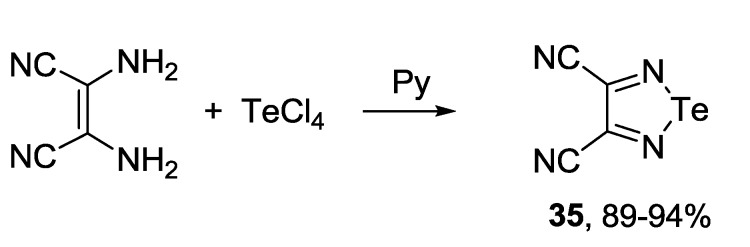
Synthesis of 3,4-dicyano-1,2,5-telluradiazole **35**.

The most common precursors for the synthesis of 1,2,5-telluradiazoles, both monocyclic and fused, are again *ortho*-diamines. 3,4-Dicyano-1,2,5-telluradiazole **35** was synthesized by the reaction of 2,3-diaminomaleonitrile with TeCl_4_ in pyridine [97,148] (Scheme 18).

**Scheme 19:**
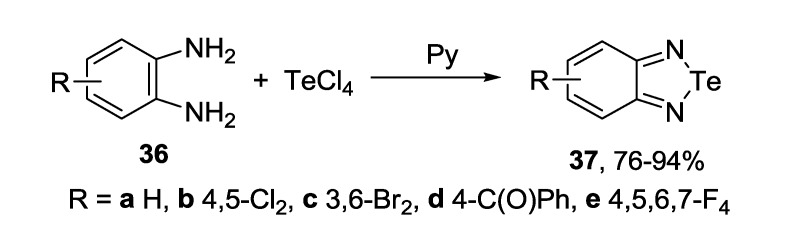
Synthesis of 2,1,3-benzotelluradiazoles **37**.

Practically the same procedure has been used for the synthesis of variously substituted benzo-fused 1,2,5-telluradiazoles (2,1,3-benzotelluradiazoles) **37** (Scheme 19). Normally, pyridine was employed as a solvent and as a base [149,150,151,152]. In the absence of organic base, 2,2-dichloro-5,6-dimethyl-2,1,3-benzotelluradiazole was obtained from 4,5-dimethyl-1,2-phenylenediamine and TeCl_4_ in 1,2-dichlorobenzene under reflux [153]. 4,6-Di-*tert*-butyl derivative of compound **37a** was prepared by reaction of 2,4,6-t-Bu_3_C_6_H_2_NHLi with Ph_2_P(NSiMe_3_)_2_Te(Cl)NPPh_2_NSiMe_3_ in toluene [154].

Attempt to prepare telluradiazole **37a** by heating a mixture of 1,2-phenylenediamine with tellurium dioxide resulted in its hydrated analogue (benzo-2-telluroxo-1,3-diazole) whereas from 4,5-dimethyl derivative of the diamine only 1-amino-3,4,7,8-tetramethylphenazine was obtained [153].

**Scheme 20:**
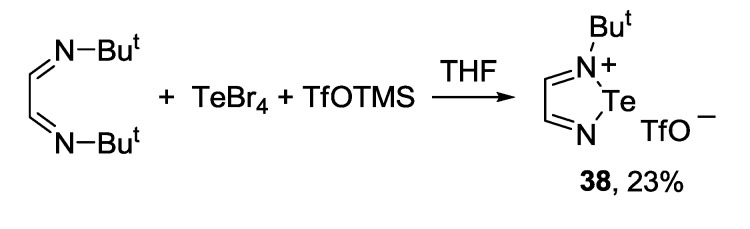
Synthesis of 2-*tert*-butyl-1,2,5-telluradiazol-2-ium triflate **38**.

A variety of 1,2,5-telluradiazolium structures was also described. For example, 2-*tert*-butyl-1,2,5-telluradiazol-2-ium triflate **38** was synthesized by condensation of 1,4-di(*tert*-butyl)-1,4-diaza-1,3-butadiene with TeBr_4_ and trimethylsilyl triflate in THF with low yield [155] (Scheme 20).

**Scheme 21:**
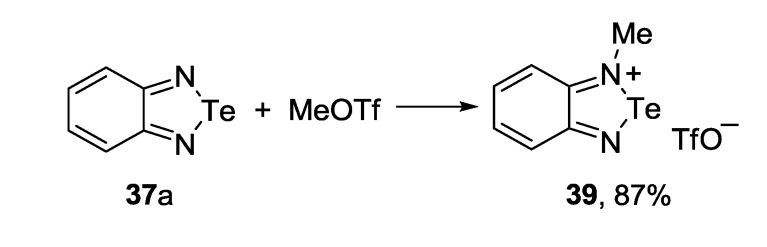
Synthesis of 2-methylbenzotelluradiazol-2-ium triflate **39**.

2-Methylbenzotelluradiazol-2-ium salt **39** was prepared by alkylation of 2,1,3-benzotelluradiazole **37a** with methyl triflate in dichloroethane [155] (Scheme 21).

**Scheme 22:**
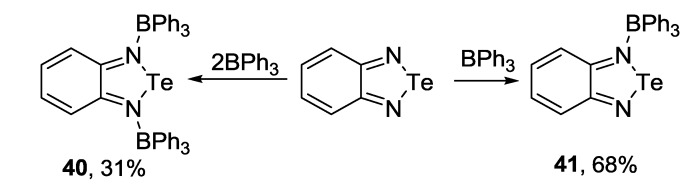
Synthesis of triphenylborane complexes of benzotelluradiazole **37a**.

Molecular complexes **40** and **41** were obtained from 2,1,3-benzotelluradiazole **37a** and triphenylborane featuring molar ratio of 2:1 and 1:1, respectively [150] (Scheme 22).

**Scheme 23:**
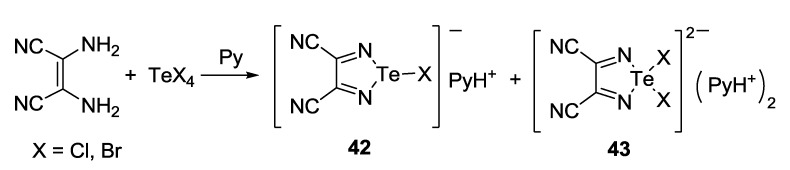
Synthesis of pyridinium salts of the hypercoordinate adducts **42** and **43**.

The synthesis of 3,4-dicyano-1,2,5-telluradiazole **35** (Scheme 18) by the reaction of 2,3-dimalononitrile with tellurium chloride and bromide is a rather complex process. It is accompanied by the formation of hypercoordinate adducts **42** and **43** (X = Cl, Br) isolated in the form of pyridinium salts (Scheme 23) [97]. When TeCl_4_ was used with triethylamine, salt of the adduct **42** with Et_3_NH^+^ was isolated. This reaction seems to be general since adducts with fluoride and iodide ions were also isolated in special experiments following by structural characterization by X-ray diffraction (XRD) [156]. According to the DFT calculations, adduct with fluoride is the most stable [88].

**Scheme 24:**
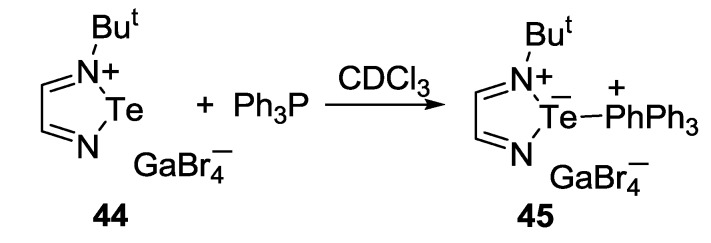
Synthesis of salt **45**.

1,2,5-Telluradiazole-based salt **45** was prepared by the reaction of salt **44** with triphenylphosphine [157] (Scheme 24).

Very recently, porphyrazines with annulated 1,2,5-telluradiazole ring(s) were synthesized [158].

### 3.4. 1,2,6-Chalcogenadiazines

Besides 1,2,5-chalcogenadiazoles, there is very interesting group of miscellaneous chalcogen-nitrogen π-heterocycles covering their isomers, 1,2,6-thia(selena)diazines and some other systems partially represented in Table 2, Table 3, Table 4, Table 5 and Table 6 and expected to be precursors of stable RAs. Some of these compounds are known for a long time [83,84,85,159,160,161,162,163,85,159] but did not attract attention within RA chemistry for various reasons. Selection of 1,2,6-thia(selena)diazines discussed below is based on their involvement in ongoing research by the authors. Te congeners of these heterocycles are unknown.

#### 3.4.1. 1,2,6-Thiadiazines

**Scheme 25:**
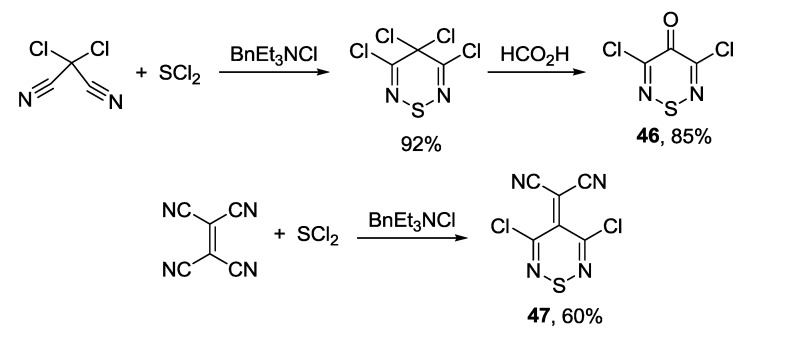
Synthesis of 4*H*-1,2,6-thiadiazines **46** and **47**.

The synthesis and reactivity of 1,2,6-thiadiazines has been reviewed [164]. Surprisingly, little is known on nonoxidized 4*H*-1,2,6-thiadiazines. Monocyclic 3,5-dichloro-4*H*-1,2,6-thiadiazin-4-one **46** and its 4-dicyanomethylene analogue 2-(3,5-dichloro-4*H*-1,2,6-thiadiazin-4-ylidene)malononitrile **47** have been prepared, the former in two steps starting from dichloromalononitrile [165] and the latter in one step from TCNE [166,167] (Scheme 25). Both are useful precursors to several polycyclic 1,2,6-thiadiazine systems.

**Scheme 26:**
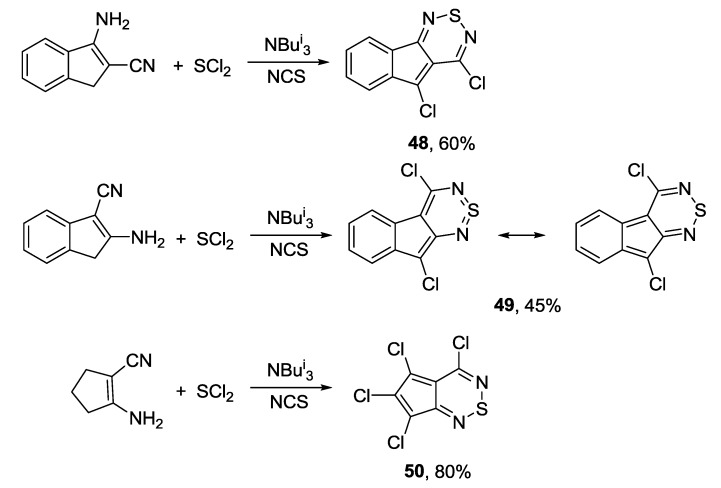
Synthesis of cyclopenta[1,2,6]thiadiazines **48**–**50**.

More recently, cyclopenta[1,2,6]thiadiazines **48**–**50** were prepared starting from cyclic enaminonitriles, sulfur dichloride, *N*-chlorosuccinimide (NCS) and triisobutylamine (Scheme 26) [168,169].

**Scheme 27:**
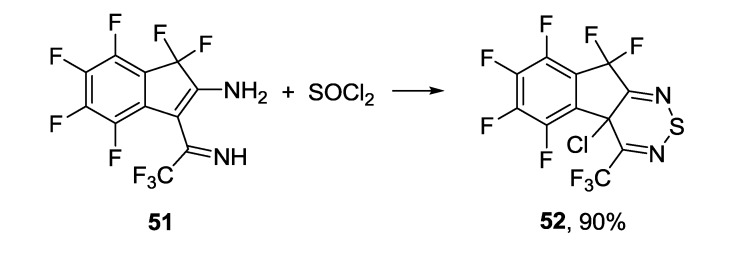
Synthesis of fluorinated dihydroindeno[2,1-c][1,2,6]thiadiazine **52**.

1,2,6-Thiadiazine ring may be constructed from enaminoimino derivatives as it was shown by the reaction of 3-(1-amino-2,2,2-trifluoroethyliden)-1,1,4,5,6,7-hexafluoro-2-iminoindane **51** with thionyl chloride (Scheme 27) [170].

**Scheme 28:**
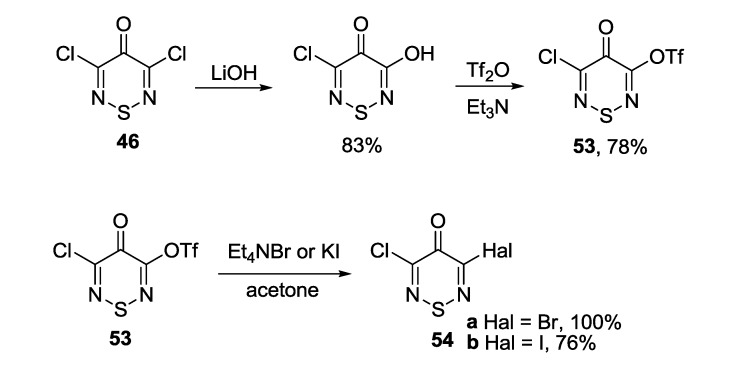
Preparation of 5-substituted 3-chloro-4*H*-1,2,6-thiadiazin-4-ones **53** and **54**.

Very recently, compound **46** was extensively used for the preparation of 3-halogen or oxy derivatives employed further for arylation of thiadiazines. 5-Trifluorosulfonate **53** was synthesized by successive treatment of **46** with LiOH in dry THF and trifluoromethanesulfonic anhydride (Tf_2_O) in the presence of triethylamine in CH_2_Cl_2_ (Scheme 28) [171]. 3-Bromo-5-chloro-4*H*-1,2,6-thiadiazin-4-one **54a** and 3-chloro-5-iodo-4*H*-1,2,6-thiadiazin-4-one **54b** were prepared in high yields from **53** by exchange reaction with Et_4_NBr and KI, correspondingly. 3-Halo-5-phenylthiadiazinones were synthesized by a similar way [172].

**Scheme 29:**
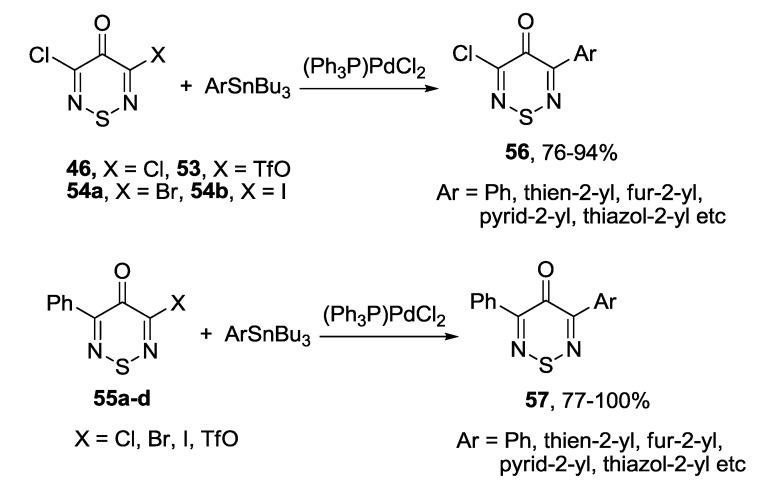
Stille reaction with 4*H*-1,2,6-thiadiazin-4-ones.

The Stille coupling was the most frequently used procedure for the preparation of (het)aryl substituted 1,2,6-thiadiazines **56** and **57** from corresponding halogen (**46**, **54**, **55a**–**c**) or triflate (**53**, **55d**) derivatives and tributylstannanes [171,172,173]. Palladium catalysts were used in these syntheses, yields were high (Scheme 29).

**Scheme 30:**
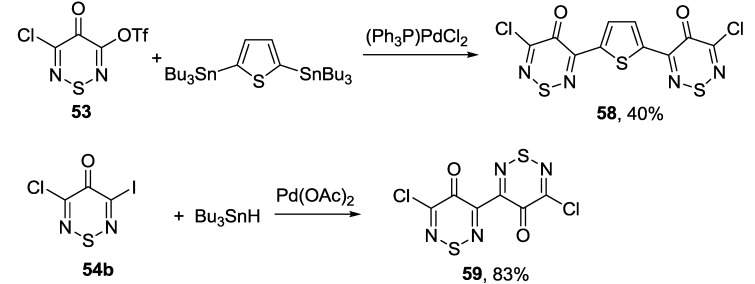
Synthesis of bis(4*H*-1,2,6-thiadiazin-4-ones).

Bis-adducts of 3-chloro-4*H*-1,2,6-thiadiazine series may be also obtained from corresponding triflate or iodo derivatives (**53** and **54b**) and 2,5-bis(tributyltin)thiophene or Bu_3_SnH which was used as a reducing agent (Scheme 30) [171].

**Scheme 31:**
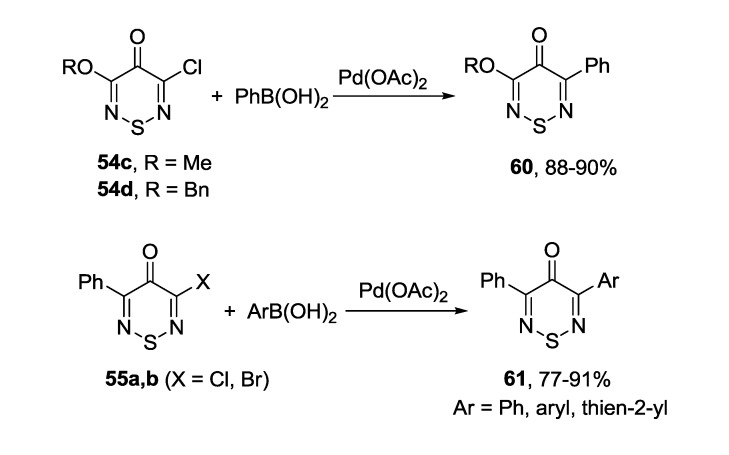
Suzuki coupling in 3-halo-4*H*-1,2,6-thiadiazin-4-ones.

The Suzuki coupling of chloro- or bromo-4*H*-1,2,6-thiadiazin-4-ones (**54c**,**d** and **55a**,**b**) with arylboronic acids in the presence of Pd(OAc)_2_ and sodium carbonate led to mono-adducts in high yields (Scheme 31) [172,173].

#### 3.4.2. 1,2,6-Selenadiazines

**Scheme 32:**
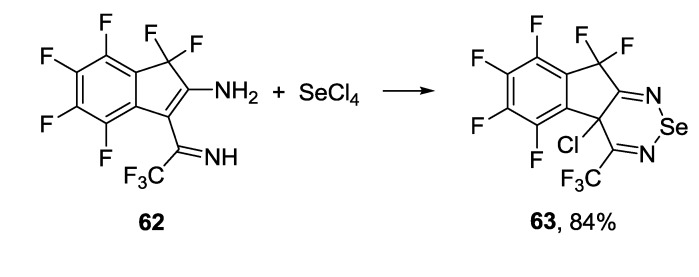
Synthesis of polyfluorinated dihydroindeno[2,1-c][1,2,6]selenadiazine **63**.

There is only one known example of the 1,2,6-selenadiazine synthesis, *i.e.*, the reaction of 3-(1-amino-2,2,2-trifluoroethyliden)-1,1,4,5,6,7-hexafluoro-2-iminoindane **62** with SeCl_4_ (Scheme 32) [174].

## 4. Charge-Transfer Complexes and Radical-Ion Salts

As mentioned above, 1,2,5-chalcogenadiazoles and some other chalcogen-nitrogen π-heterocycles featuring positive EA form a new group of efficient electron acceptors. Electron transfer between donor (D) and acceptor (A) molecules can be incomplete, *i.e.*, leading to CT complexes D^δ+^A^δ−^, or complete, *i.e.*, leading to radical-ion salts D^+^A^−^. 1,2,5-Chalcogenadiazoles are involved in both types of this interaction [70,71,72,73,74,76,77,78,79,89,93,95,96].

### 4.1. Synthesis and Characterization

#### 4.1.1. Charge-Transfer Complexes

One of the most well known organic electron donors is tetrathiafulvalene (TTF), IUPAC name: 2,2’-bis(1,3-dithiolydene) [175]. Its 0/+1 electrochemical potential is 0.33 V [18], and the HeI UPS first vertical IE is ~6.8 eV [176,177]. With electron acceptors, TTF, as well as its various derivatives, form numerous CT complexes and radical-ion salts. In the majority of cases, TCNE, TCNQ and their derivatives were used as electron acceptors. Many of compounds prepared display electrical conductivity and superconductivity as well as interesting magnetic properties [4,14,95,175,178].

**Figure 4:**
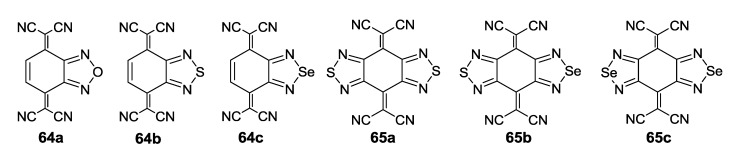
TCNQ-fused 1,2,5-chalcogenadiazoles and their O congener.

Initially, the 1,2,5-chalcogenadiazoles (chalcogen: S, Se) and their O congener fused with TCNQ (Figure 4) were used in the synthesis of CT complexes and radical-ion salts [95,96]. This was motivated by the assumption that the heterocyclic moieties will enforce intermolecular interactions suppressing the metal-insulator transition typical of many TCNQ-based complexes and salts.

A series of TCNQ derivatives fused with one 1,2,5-oxa- and- thia(selena)diazole unit (**64a**–**c**) was synthesized and their reduction potentials (E_p_, Table 7) were found to be positive, although for **64b**,**c** the E_p_ values were somewhat lower than that for TCNQ. These heterocyclic TCNQ derivatives gave a series of CT complexes with TTF derivatives [95].

The TCNQ derivatives fused with two 1,2,5-thia(selena)diazole units (**65a**–**c**) have small negative reduction potentials (Table 7). Formation of their 1:1 CT complexes with *para*-xylene, durene, 2,6- and 2,7-dimethylnaphthalene has been observed and their crystal structures have been studied in detail [96]. Later, a number of CT complexes of **65a** with tetrathiatetracene (TTT; IUPAC name: tetraceno[5,6-cd:11,12-c’d’]bis[1,2]dithiole), TTF and its derivatives, and aromatic amines were prepared [179,180].

**Scheme 33:**
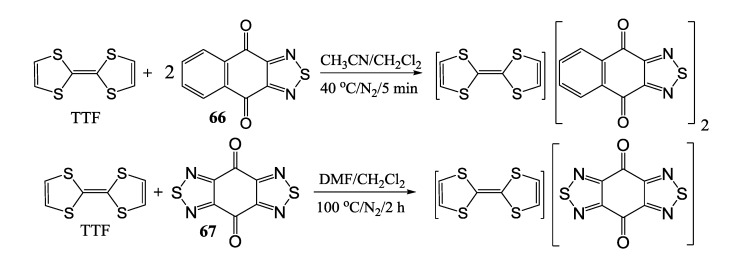
CT complexes between TTF and quinone-fused 1,2,5-thiadiazoles **66** and **67**.

In addition, *para*-naphtho- and -benzoquinone derivatives fused with 1,2,5-thiadiazoles **66** and **67** were used in the synthesis of CT complexes with TTF. Reaction of TTF with **66** gave 1:2 complex whereas that with **67** the 1:1 complex [98] (Scheme 33).

**Figure 5:**
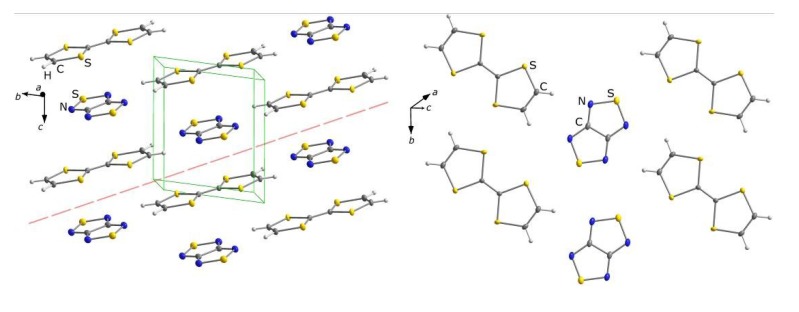
XRD structure of CT complex TTF**·22**. Layers containing flat D and A molecules are extended in the directions marked by the dashed line (left). Each layer contains the alternated rows of D and A molecules propagated along the *b* axis (right).

**Figure 6:**
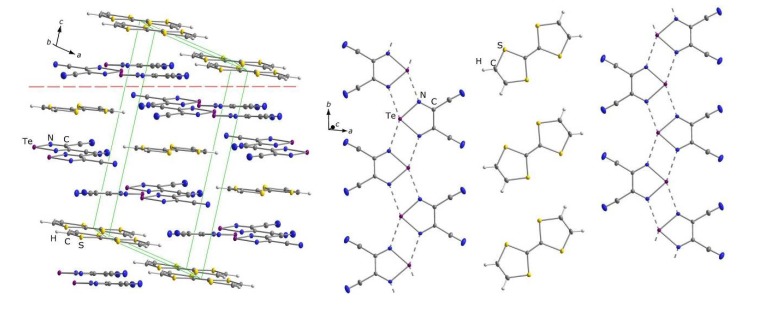
XRD structure of CT complex TTF**·35_2_**. Layers containing flat D and A molecules are extended in the directions marked by the dashed line (left). Each layer contains chains propagated along the *b* axis. The chains are composed of coordinatively bound molecules of **35** alternating with separate molecules of TTF (right).

Recently, it was recognized that 1,2,5-chalcogenadiazoles themselves can serve as effective electron acceptors (Section 2). With TTF, it was discovered that [1,2,5]thiadiazolo[3,4-c][1,2,5]thiadiazole **22** and 3,4-dicyano-1,2,5-telluradiazole **35** form EPR-silent CT complexes TTF·**22** and TTF·**35_2_**, respectively, in the latter case despite the fact that the initial molar ratio of components in reaction solution was 1:1 [89]. The structures of both complexes were confirmed by XRD (Figure 5 and Figure 6). The B97-D functional taking into account long-range dispersion interactions was employed in the calculations of the formation of CT complexes TTF**·22** and TTF**·35_2_**. This method is well suited for the description of weakly bonded complexes (e.g., D-A, van der Waals, and H-bonded complexes) [181]. The def2-TZVP basis set with diffuse basis functions and ECP were used for Te [182]. According to the calculations, electron transfer from TTF is 0.24e onto molecule of **22** and 0.39e onto two molecules of **35**. For complex TTF**·22**, the QTAIM calculations identified five bond critical points (BCPs) for C…N, S…N, and S…S bonding between its components. All the BCPs were characterized by low electron density (*ρ_BCP_* = 0.5–1.1 × 10^−2^ a.u.) and positive values of the Laplacian (▽^2^*ρ_BCP_* = 1.6–2.6 × 10^−2^ a.u.), the latter being typical of the closed-shell D…A interactions [89]. Experiments with stronger electron donor TTT featuring 0/+1 electrochemical potential of 0.15 V [183] are in progress [184].

#### 4.1.2. Radical-Ion Salts

Radical-ion salts of the heterocycles under discussion can be homospin, *i.e.*, only anion is paramagnetic, and heterospin, *i.e.*, both ions are paramagnetic. With 1,2,5-chalcogenadiazoles as starting materials, both types of radical-ion salts were synthesized [70,71,72].

The homospin, S = 1/2, RA salts of 1,2,5-chalcogenadiazoles were prepared with a number of reducing agents covering elemental potassium [74] (used previously for EPR experiments [57,58,59,60,61,62,63,64]), thiophenolate and its Se congener [77,78], tetrakis(dimethylamino)ethylene (TDAE) [76], and cobaltocene (CoCp_2_) [75].

**Scheme 34:**
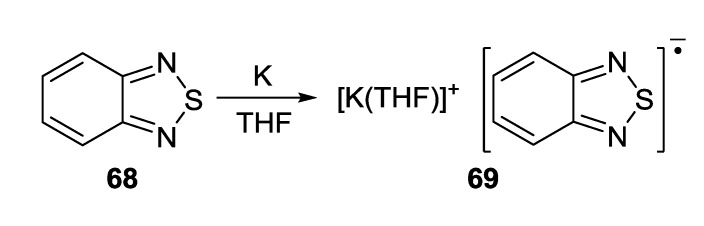
Synthesis of homospin RA salt **69**.

**Figure 7:**
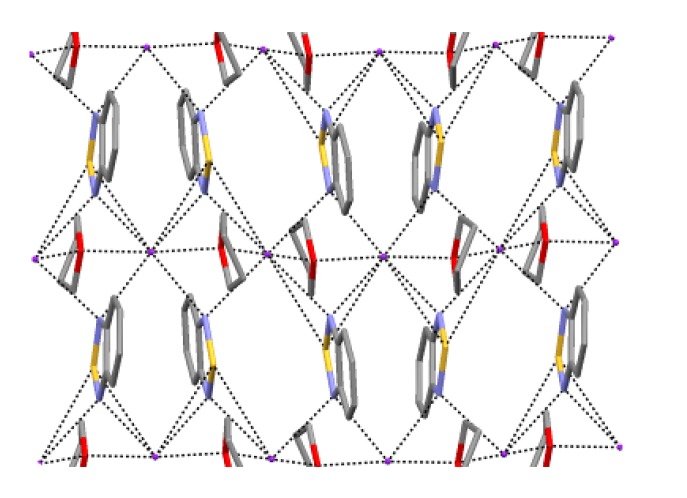
XRD structure of RA salt **69**.

With elemental potassium and in THF, 2,1,3-benzothiadiazole **68** was transformed into RA salt isolated as [K(THF)][**68**] (**69**) (Scheme 34, Figure 7) [74].

**Scheme 35:**
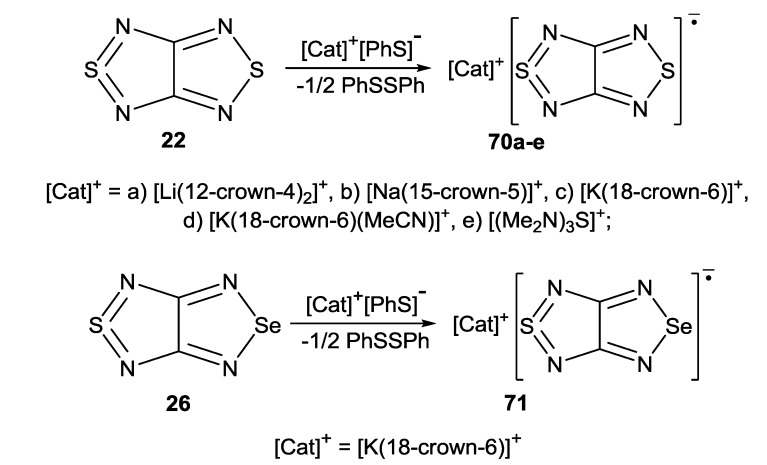
Synthesis of homospin RA salts **70** and **71**.

**Figure 8:**
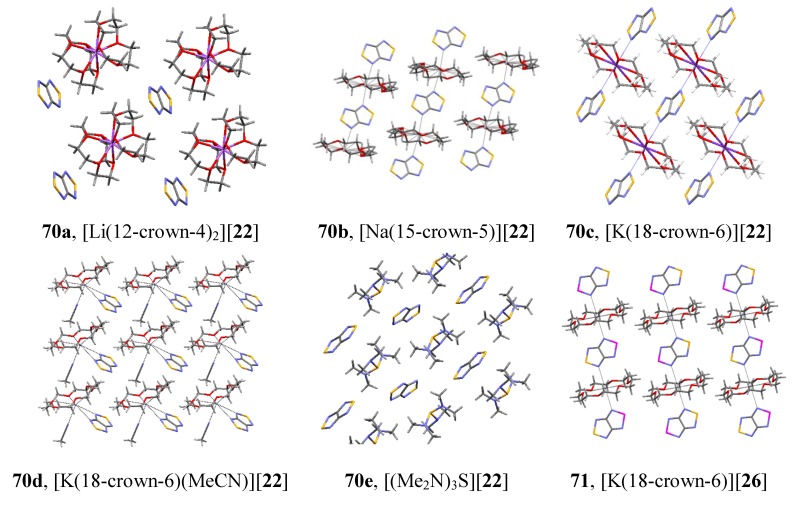
XRD structures of the RA **70a**–**e** and **71** obtained by reduction of heterocycles **22** and **26** with thiophenolate.

Bicyclic chalcogenadiazoles **22** and **26** were reduced with thiophenolate. This approach made it possible to obtain RA salts **70** and **71** with diamagnetic cations of early alkali metals (Li^+^, Na^+^, K^+^) encapsulated into corresponding crown ethers as well as the salt with (Me_2_N)_3_S^+^ cation (Scheme 35). The structures of all salts were confirmed by XRD (Figure 8) and their paramagnetic character by solid-state and solution EPR in combination with DFT calculations [77,78].

**Scheme 36:**
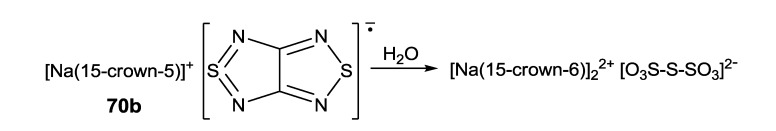
Heterogeneous hydrolysis of RA salt **70b**.

**Figure 9:**
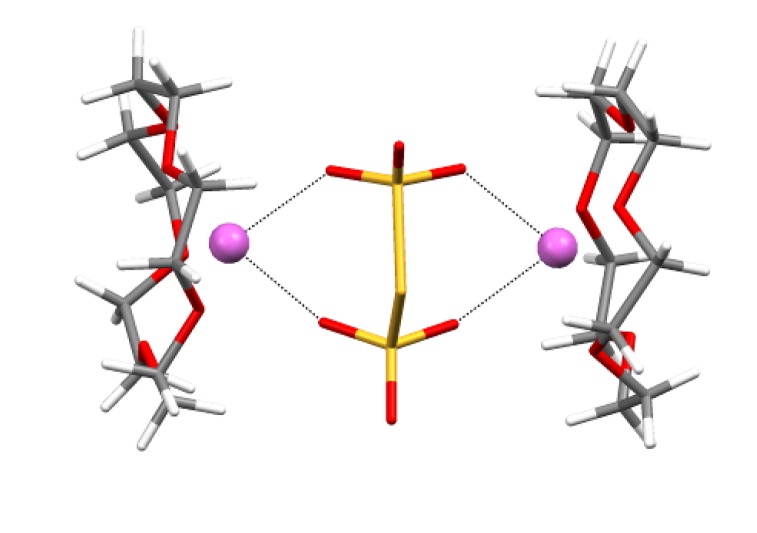
XRD structure of salt [Na(15-crown-5)]_2_[S_3_O_6_] obtained by hydrolysis of RA salt **70b**.

The salts prepared are thermally stable but air-sensitive. Their decomposition, however, is not a fast process. Specially designed heterogeneous hydrolysis of crystalline salt **70b** with saturated water vapor at ambient temperature showed that it proceeds quite slowly and results in an unexpected formation of trithionate salt (Scheme 36, Figure 9) [185].

**Scheme 37:**
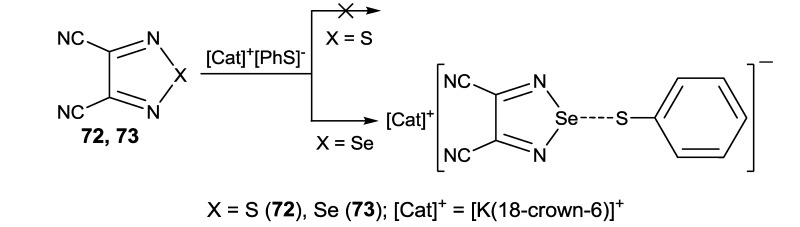
Hypercoordination of thiophenolate to Se center of compound **73**.

**Figure 10:**
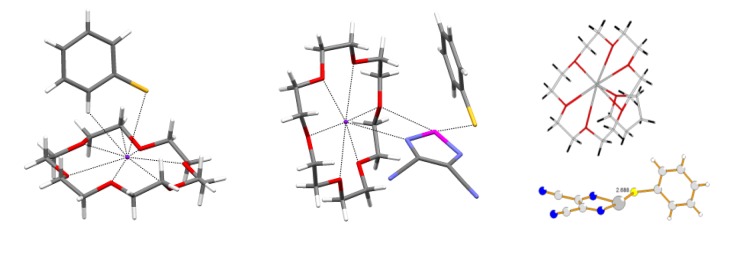
XRD structures of reaction products from thiophenolate and compounds **73** (middle) and **35** (right; solvate with THF) in comparison with that of [K(18-crown-6)]^+^[PhS]^−^ (left).

The thiophenolate-based approach has, however, a limited scope since, for example, the anion does not react with 3,4-dicyano-1,2,5-thiadiazole **72** and 2,1,3-benzothiadiazole **68** [186]. On the other hand, the reaction of thiophenolate with 3,4-dicyano-1,2,5-selenadiazole **73** and 3,4-dicyano-1,2,5-telluradiazole **35** gave products of its hypercoordination at the Se and Te centers of the heterocycles, respectively (Scheme 37, Figure 10) [156,186]. It should be noted that similar hypercoordination at the Te center of compound **35** was observed for halides X^−^ (X = F, Cl, Br, I) [97,156]. This previously unknown type of reactivity may be rather general for 1,2,5-chalcogenadiazoles containing heavier chalcogens. According to the XRD data, in all studied cases the length of the hypercoordinate bond is *ca.* 0.3–0.5 Å longer than the sum of corresponding covalent radii but *ca.* 1–1.2 Å shorter than the sum of corresponding van der Waals (VdW) radii. For example, in the hypercoordinate product shown in Scheme 36 and Figure 10 (middle) the Se-S distance is 2.722 Å as compared with the sum of the covalent radii of these atoms of 2.25 Å and the sum of their VdW radii of 3.70 Å. For derivatives of Ph-Se-S-Ph with known XRD structures the Se-S bond length varies in the range 2.20–2.22 Å, for neutral derivatives of 3-coordinated Se atom in the range 2.50–2.58 Å, and for derivatives of the 4-coordinated Se atom in the range 2.78–3.05 Å ([186] and references therein). In the hypercoordinate product featuring the Te-S bond (Figure 10, right), its length is 2.688 Å whereas the sums of the corresponding covalent and VdW radii are 2.43 and 3.86 Å, respectively [156].

According to the NBO calculations, in all cases the hypercoordinate bond is formed via negative hyperconjugation of a lone-pair orbital of X^−^ (X = PhS, Hal) with antibonding σ*-MO of the chalcogen-nitrogen bond of heterocycle. This description agrees with the Alcock model suggested previously for secondary bonding interactions between atoms of heavy p-block elements and atoms with electron lone pairs [97,156,186].

The dichotomy between reduction to RA and hypercoordination to heavier chalcogen atom should be taken into account in further work in the field.

**Scheme 38:**
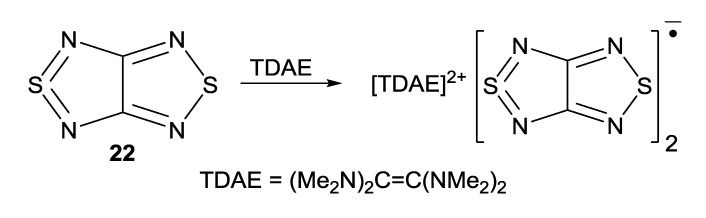
Synthesis of homospin RA salt [TDAE][**22**]_2_.

**Figure 11:**
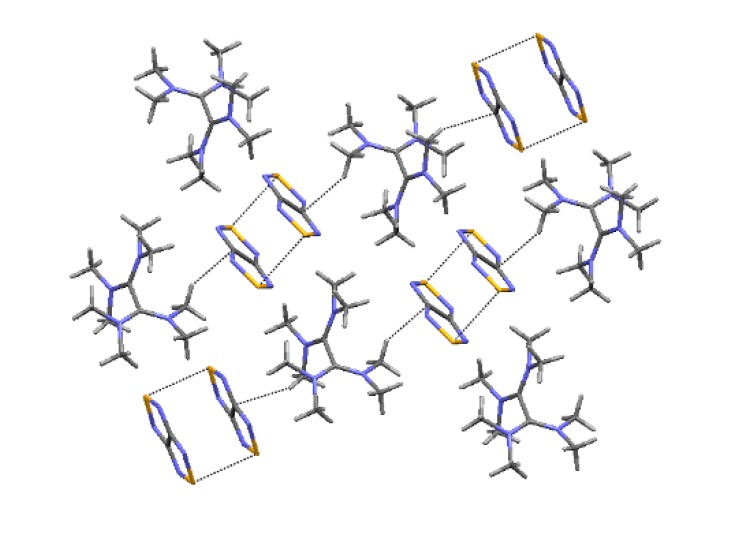
XRD structure of RA salt [TDAE][**22**]_2_.

Interaction of compound **22** with TDAE gave homospin RA salt [TDAE][**22**]_2_ (Scheme 38) which was EPR-active in solution but silent in the solid state. According to XRD data (Figure 11), in the crystal the RAs form centrosymmetric π-dimers featuring interplanar separation of 3.25 Å whereas the sum of the VdW radii of two S atoms is 3.60 Å. These diamagnetic dimers are stable only in the solid state and dissociate in both the solution (EPR spectroscopy) and gas phase (DFT calculations) [76].

**Scheme 39:**
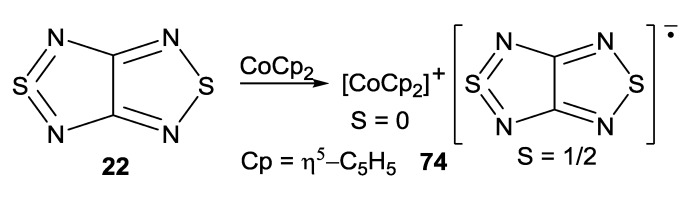
Synthesis of homospin RA salt [CoCp_2_][**22**].

**Figure 12:**
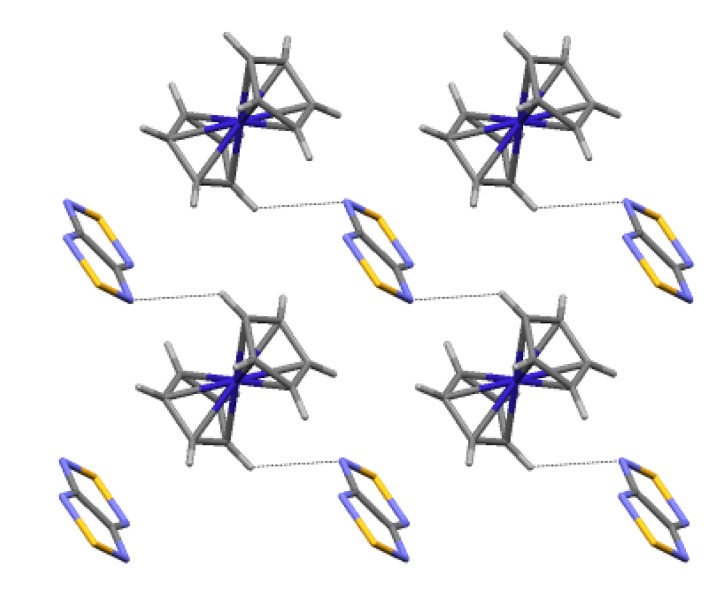
XRD structure of RA salt **74**.

With cobaltocene, heterocycle **22** gave homospin RA salt [CoCp_2_][**22**] (**74**) (Scheme 39, Figure 12) which is EPR-active in both the solid state and solution [75].

**Scheme 40:**
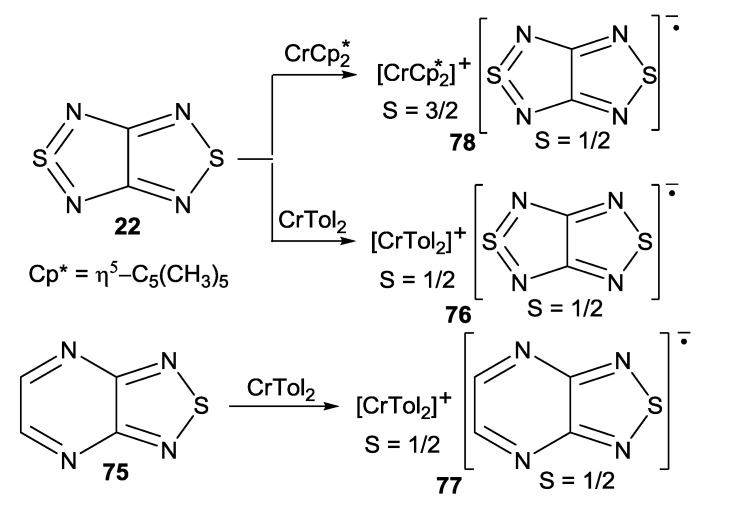
Synthesis of heterospin RA salts **76**–**78**.

**Figure 13:**
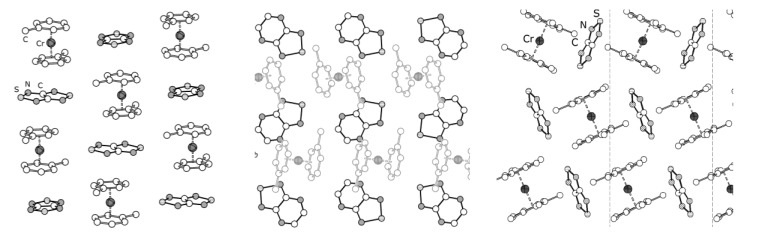
XRD structures of heterospin RA salts **76** (left), **77** (middle) and **78** (right).

The heterospin, S_1_ = S_2_ = 1/2 and S_1_ = 3/2, S_2_ = 1/2, RA salts of 1,2,5-chalcogenadiazoles **22** and **75** were prepared with reducing agents bis(toluene)chromium(0) (CrTol_2_) and decamethylchromocene (CrCp*_2_), respectively, as [CrTol_2_][**22**] (**76**), [CrTol_2_][**75**] (**77**) and [CrCp*_2_][**22**] (**78**) (Scheme 40, Figure 13). Whereas salts **76** and **77** are EPR-active in both the solid-state and solution, salt **78** is EPR-silent in these states of aggregation due to the substantial zero-field splitting and fast relaxation of the cation provoking fast relaxation of the RA [71,73]. In solution the salts exist most likely in the form of ion pairs which is typical of the discussed RAs [57,58,59,60,61,62,63,64].

**Scheme 41:**
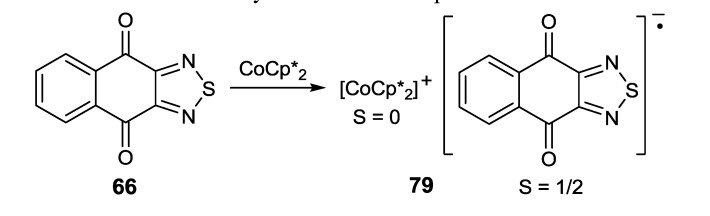
Synthesis of homospin RA salt **79**.

The naphtho[2,3-c][1,2,5]thiadiazole-4,9-dione **66** was reduced by CoCp*_2_ and CoCp_2_, and two types of RA salts, [CoCp*_2_][**66**] (**79**) and [CoCp_2_]_2_[**66**]_3_ (**80**), respectively, were isolated [99]. Analysis of the XRD structure of the solvate **79·**3CH_2_Cl_2_ confirmed the complete CT with formation of the RA of **66** (Scheme 41). The EPR spectrum of the latter was recorded for CH_2_Cl_2_ solution of salt **79**.

With salt **80**, situation is more complicated. On the basis of charge balancing rules and careful analysis of the C-O bond distances, it was concluded that the unit cell of **80** is comprised of two cations [CoCp_2_]^+^, one RA of **66**, and one bimolecular RA unit (**66**)_2_^•−^. In the latter, the unpaired electron and the negative charge are delocalized equally across two molecules of **66** [99].

**Scheme 42:**
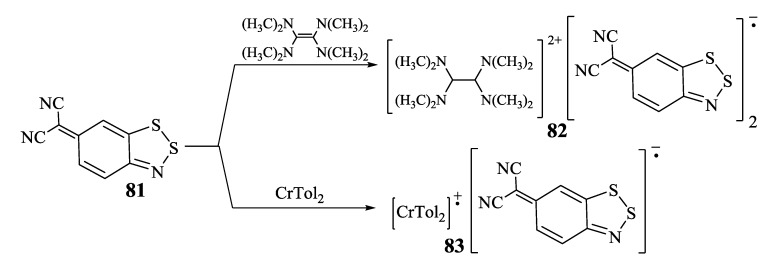
Synthesis of RA salts **82** and **83**.

Very recently [80], a new type of sulfur-nitrogen π-heterocycles represented by (6*H*-1,2,3-benzodithiazol-6-ylidene)malononitrile **81** was electrochemically and chemically with TDAE and CrTol_2_ (Scheme 42) reduced into the RA. Electrochemically generated RA and its salts [TDAE][**81**]_2_ (**82**) and [CrTol_2_][**81**] (**83**) were characterized by solution EPR. According to the EPR data, salt **82** is homospin S = 1/2 paramagnet in both the solid state and MeCN solution. Solid-state paramagnetism of salt **82** indicates that RAs do not form diamagnetic π-dimers. Salt **83** is heterospin, S_1_ = S_2_ = 1/2, since both paramagnetic ions were detected in its THF solution [80].

**Figure 14:**
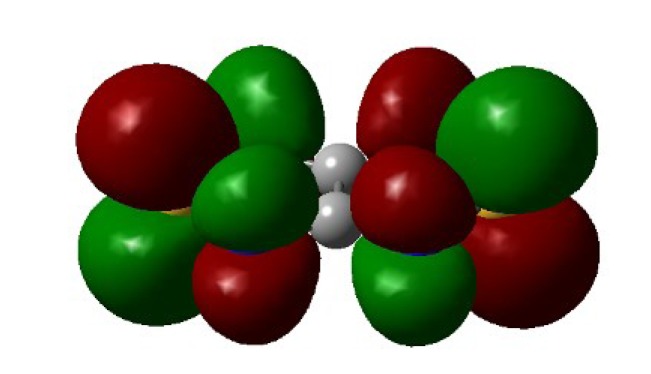
The π-SOMO of RA of compound **22**.

Electronic structure and EPR spectra of RAs obtained by electrochemical and chemical reduction of chalcogen-nitrogen π-heterocycles were analyzed using results of the DFT, HF and, in some cases, post-HF methods [70,71,72,73,74,75,76,77,78,79,80]. For all RAs under study, the SOMO is a π-type MO. Figure 14 displays the SOMO of the typical representative, *i.e.*, RA of compound **22**. This π-SOMO is antibonding for bonds SN and nonbonding for bonds CN.

Multi-configuration calculations by the CASSCF method revealed an unusual electronic structure of π-dimers of RAs for the salt [TDAE][**22**]_2_ (see Figure 11). It was found that the major contribution (~80%) to the wave function of the singlet ground state of the dimer comes from the electronic configuration with two electrons occupying the bonding MO composed of weakly interacting SOMOs of the RAs. However, the contribution of the biradical component to this state is rather large (~20%) [76].

**Figure 15:**
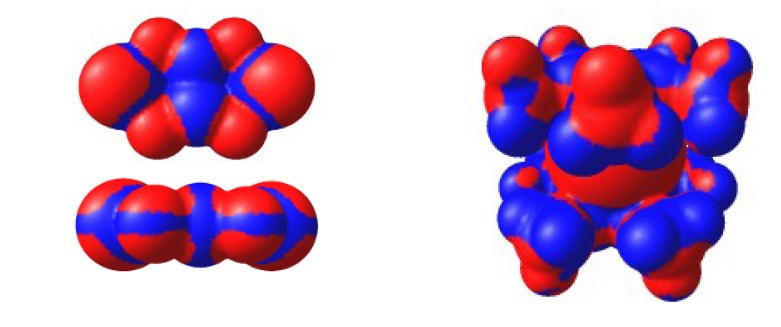
Spin density (ρ) distribution over the VdW surface of RA of compound **22** (left) and cation [CrCp*_2_]^+^ (right) from the UB3LYP/6-31+G(d) calculations. Color code: red, ρ > 0; blue, ρ < 0.

The DFT-calculated spin density distribution in the RAs studied shows that the density on their VdW surfaces is mainly positive with only small islands of negative values in the vicinity of the C-C bond of 1,2,5-chalcogenadiazole ring (for example, see Figure 15) [56,73]. Therefore, contacts between the like spin density regions are most probable for neighboring RAs in the crystals of homospin salts. This should lead to antiferromagnetic (AF) exchange interactions between RAs. If two paramagnetic species in heterospin salts mainly contact each other in the regions of unlike spin density, ferromagnetic (FM) interactions are possible [187,188,189,190,191,192,193,194].

### 4.2. Electrical and Magnetic Properties

#### 4.2.1. Charge-Transfer Complexes

**Figure 16:**
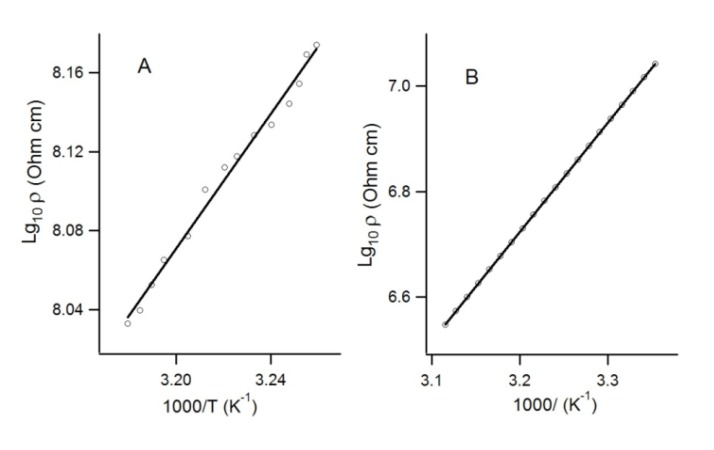
Temperature dependence of the resistance of single crystals of CT complexes TTF**·22**(**A**) and TTF**·35_2_** (**B**): circles—experiment, straight lines—the best linear fit.

The standard two-contact method was used to measure the single crystals resistivity of the CT complexes TTF**·22** and TTF**·35_2_**. The temperature dependence of resistance was recorded in the temperature range 300–320 K (Figure 16). As follows from Figure 16, both complexes revealed low-gap semiconductor behavior with activation energy ~0.34 eV for TTF**·22** and 0.40 eV for TTF**·35_2_**.

For both CT compounds, the absolute conductivity showed noticeable increase upon white-light irradiation (0.5–1.5 sun intensity) which is congruent with the D-A system’s ability to efficiently separate the generated charges. Although the relative increase in conductivity was small, this was attributed to the comparatively large dark currents arising from these low-gap semiconductors [89].

#### 4.2.2. Radical-Anion Salts

Molar magnetic susceptibilities (χ) of a series of RA salts under discussion were measured in a wide temperature range 2–300 K. For the homospin salt [TDAE][**22**]_2_ characterized by centrosymmetric π-dimers of the RAs in the XRD structure, the magnetic susceptibility is temperature-independent and equal to zero after subtracting the diamagnetic contribution [76].

For all other studied RA salts, the magnetic susceptibility significantly increases with decreasing temperature indicating their paramagnetic character [71,72,73,74,75,76,77,78,79,80].

**Figure 17:**
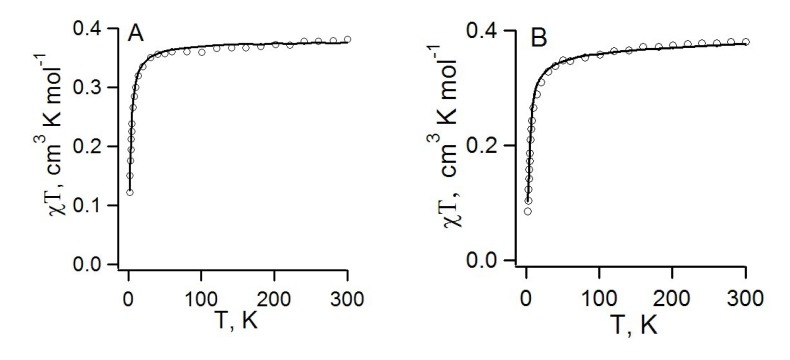
Experimental χ(T)T dependences (circles) and their theoretical approximation (solid curves) using Bonner-Fisher uniform chain model with *J* = −1.22 cm^−1^ (**A**, salt **70c**, r = 0.9997) and *J* = −1.65 cm^−1^ (**B**, salt **71**, r = 0.997).

Experimental χ(T) dependences for RA salts **70b**,**c** with alkali metal cations were analyzed using phenomenological analytical temperature dependences [78]. For both salts, AF exchange interactions were observed, with low Neel temperatures (Table 8). For salt **70c**, the Bonner-Fisher uniform chain model [190] perfectly describes the χ(T) in the whole temperature range (Figure 17A). For salt **70b**, the alternating chain model [190] led to reasonable agreement with experiment. Experimentally determined parameters of the AF exchange interactions for these and some other homospin RA salts are presented in Table 8.

**Table 8:** Experimental and calculated parameters (*J*) of pair exchange interactions and Neel temperature (*T_N_*) for a series of homospin RA salts.

RA salt	*T_N_*, K	*J*, cm^−1^		Refs.
		Experiment	Calculation	
**69**, [K(THF)][**68**]	17	−13.5, 7.2, −2.9	−8.1, 7.2, 3.2, −1.0; *^a^* −9.0, 7.3, 1.9, −0.05; *^b^* −9.0, 4.8, −1.9, ~0; *^c^*	[74,88]
**70b**, [Na(15-crown-5)][**22**]	8	−3.42, −1.12	-	[78]
**70c**, [K(18-crown-6)][**22**]	< 2	−1.22	−2.13, *^a^* −1.62 *^b^*	[77,78,88]
**71**, [K(18-crown-6)][**26**]	< 2	−1.65	−8.08, −4.02, −1.69 *^a^* <*J*> = 3.87	[77]
**74**, [CoCp_2_][**22/**]	10	4.4 ± 0.6	−2.8 *^a^*, −4.4 *^b^*	[75,88]

*^a^* UB3LYP/6-31+G(d). *^b^* UB3LYP/TZVP. *^c^* Two K^+^ cations were taken into account in the UB3LYP/TZVP calculations.

To support application of the Bonner-Fisher uniform chain model in the case of salt **70c**, calculations of the exchange interactions for the pairs of neighboring RAs were performed. Five unique pairs of first-nearest neighbors were found in the crystal structure of salt **70c** [77]. Spin-unrestricted broken-symmetry approach [191,192,193,194,195] at HF, MP2 and B3LYP levels of theory was employed for the calculations. The calculated *J* value for one pair of RAs of **22** was at least one order of magnitude larger than other *J* values. Moreover, calculations supported the assumption that in the salt **70c** the exchanged-coupled RAs form uniform chains. In addition, the data of Table 8 demonstrate that the *J* value calculated at the UB3LYP/TZVP level (−1.62 cm^−1^) is in very good agreement with experimentally determined value −1.22 cm^−1^ (the accuracy is about 30%).

**Figure 18:**
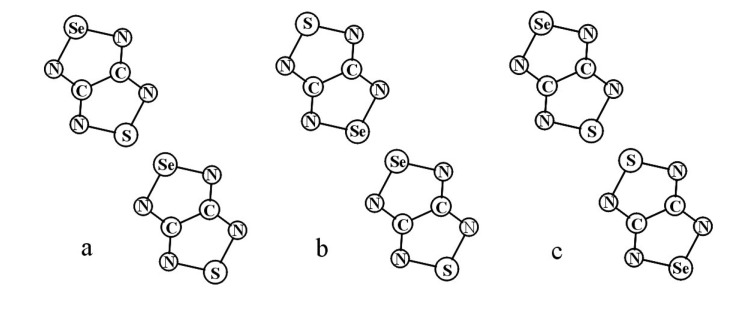
Different orientations of RAs of **26** in their selected pair for salt **71**.

The case of salt **71** is rather complicated since its structure is disordered. There are three different mutual orientations of the RAs of **26** in the selected pairs; the *J* value strongly depends on the orientation and changes from −8.08 cm^−1^ for pair *b* to −1.69 cm^−1^ for pair *c* (Table 8, Figure 18). Taking into account statistics of contacts, the average value is predicted to be −3.87 cm^−1^. The latter value is about 80% larger than *J* obtained for salt **70c**. Calculations also suggested that exchange-coupled RAs of salt **71** form infinite chains. However, these chains are characterized by three statistically distributed *J* values. Nevertheless, the Bonner-Fisher uniform chain model very well describes the χ(T) of salt **71** in the whole temperature range (Figure 17B). The experimentally obtained *J* value is about 30% larger than that for salt **70c**. This observation agrees qualitatively with the difference in the calculated *J* values [77].

**Figure 19:**
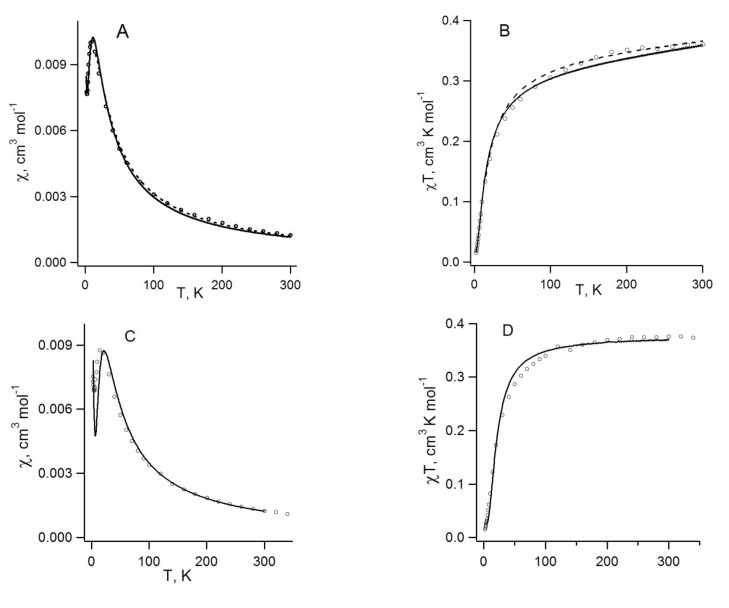
Experimental (circle) and theoretical (curves) temperature dependences of the molar magnetic susceptibility χ (**A**, **C**) and product χT (**B**, **D**) for salts **74** (A, B) and **69** (C, D). Theoretical curves: high-temperature series expansion fit (A, B; dashed curves) and calculations using the Van Vleck equation for a [3 × 4] grid of spins 1/2 (A–D, solid curves).

**Figure 20:**
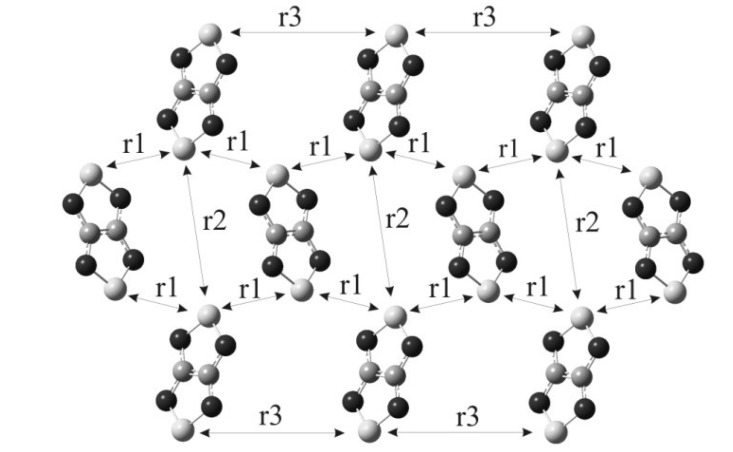
The layer of RAs of **22** in the XRD structure of salt **74** ([CoCp_2_][**22**]; r1-r3—the shortest distances between the S atoms of RAs in the unique pairs).

For RA salt **74** ([CoCp_2_][**22**]), the temperature dependence of its magnetic susceptibility has a maximum at 9.7 ± 0.5 K indicating AF ordering of the spin system (Figure 19A). The magnetic motif of salt **74** was also analyzed in terms of the pair exchange integrals (*J*) calculated similar to the case of **70c** and **71**. The magnetic motif was found to be two-dimensional (2D) and close to the S = 1/2 square lattice AF Heisenberg model (Figure 20). The analysis of the χ(T) dependence was performed using the low- and high-temperature series expansions available for this model [196,197,198,199]. The exchange interactions between RAs of **22** (with S…S distance r1, Figure 20) were estimated to be *J* = −4.2 ± 0.7 cm^−1^. With the general form of the Van Vleck equation [187,195,200] for a [3 × 4] grid of spins 1/2, the best fit of the experimental χ(T) was achieved with *J* = (−4.2)–(−5.0) cm^−1^. Experimentally determined value *J* ~ −5 cm^−1^ is in perfect agreement with *J* = −4.4 cm^−1^ calculated at the UB3LYP/TZVP level of theory.

Amongst investigated homospin RA salts, most complicated magnetic motif was predicted for **69** ([K(THF)][**68**]) [74,88]. Neglecting small *J* values (*J* < 0.2 cm^−1^) leads to the 2D magnetic motif with both FM and AF interactions (Table 8). A reasonable agreement between experimental and theoretical temperature dependences was achieved with *J* values which are one and a half times higher than calculated ones (Table 8; Figure 19, C and D).

Thus, magnetic motifs of homospin RA salts under study varies from a simple 1D motif consisting of infinite uniform chains (salt **70c**) to complex 2D motif with both FM and AF interactions (salt **69**). However, all these salts are AF materials with low Neel temperatures (T_N_ ≤ 17 K) [74,75,76,77,78].

**Figure 21:**
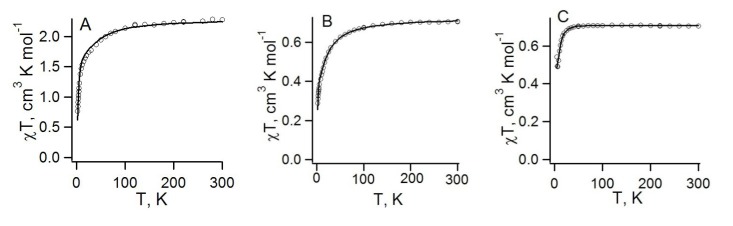
Experimental temperature dependences (circles) of the products χT in the temperature range 2−300 K for salts **78** ([CrCp*_2_][22], **A**), **76** ([CrTol_2_][**22**], **B**), and **77** ([CrTol_2_][**75**], **C**). The fitting parameters are as follows: **78**, *J*_1_ = −40 ± 9 and *J*_2_ = −0.58 ± 0.03 cm^−1^; **76**, *J*_1_ = −5.77 and *J*_2_ = −0.84 cm^−1^; **77**, *J*_1_ = 9.72 and *J*_2_ = −7.96 cm^−1^ (oversimplified model consisting of two types of anion…cation pairs).

More interesting properties might be expected for heterospin RA salts with [CrCp*_2_]^+^ (S = 3/2) and [CrTol_2_]^+^ (S = 1/2) paramagnetic cations (salts **76**–**78**, Scheme 39). For all of these salts, the temperature dependences of the product χT are typical of antiferromagnets (Figure 21). However, the salts have significantly different magnetic motifs. To calculate the pair exchange interactions, both CASSCF (or CASSCF/NEVPT2) method and spin-unrestricted broken-symmetry DFT approaches were employed [73,74].

For heterospin salt **78**, analysis of the experimental χT temperature dependence (Figure 21A) based on the CASSCF and DFT calculations revealed only AF interactions: significant between RAs of **22** (−40 ± 9 cm^−1^), weak between [CrCp*_2_]^+^ cations (−0.58 ± 0.03 cm^−1^), and very weak between the RAs and cations. Thus, magnetic motif of this salt is very simple and consists of the AF-coupled pairs of RAs and AF-coupled pairs of cations. Experimentally determined *J* values are in good agreement with calculations (Table 9).

**Figure 22:**
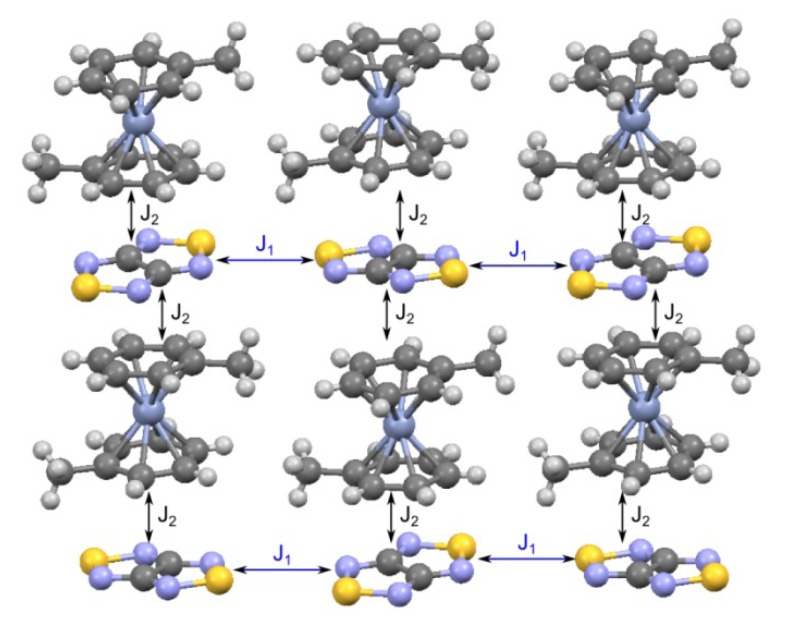
Simplified magnetic motif of heterospin RA salt **76**.

For salts **76**, calculations predict rather simple 2D magnetic motif (Figure 22) with two types of AF interactions: between RAs (*J*_1_) and between RAs and cations (*J*_2_). The theoretical modeling of the χT temperature dependence was based on this magnetic motif and led to very good agreement with experiment (Figure 21B). Experimentally determined *J* values were in reasonable agreement with experiment (Table 9).

The magnetic motif of the salt **77** is much more complex, mainly due to a structural disorder of the RAs of **75**. This 3D motif can be characterized by at least seven *J* parameters of both signs (FM and AF interactions, Table 9). To simulate correctly the χT temperature dependence of this material is intractable problem. However, even oversimplified model composed of two types of exchange-coupled pairs (*J*_1_ = 9.72 and *J*_2_ = −7.96 cm^−1^) perfectly describes experiment (Figure 21C). This is in qualitative agreement with results of calculations predicting both FM and AF interactions in the anion…cation pairs (Table 9).

**Table 9:** Experimental and calculated parameters (*J*) of the pair exchange interactions and Weiss temperatures (*θ*_W_) for a series of heterospin RA salts.

RA salt	*θ*_W_, K	*J*, см^−1^		Refs.
		Experiment	Calculations	
**78**, [CrCp*_2_][**22**]	−9.8	−40 ± 9, −0.58 ± 0.03	−61, −0.28; *^a^* −15.3, −0.08 *^b^*	[73]
**76**, [CrTol_2_][**22**]	−7.1 K	−5.77, −0.84	−8.25; *^a^* −7.45, −1.55 *^c^*	[71]
**77**, [CrTol_2_][**75**]	0.4 K	9.72, *^d^*−7.96 *^d^*	2.5, 0.7, −2.4, −8.3; *^c^*^,*e*^ −0.60, −1.70, −2.20 *^c^*^,*f*^	[71]

*^a^* UB3LYP/6-31+G(d). *^b^* CASSCF(10,10)/6-31+G(d). *^c^* NEVPT2. *^d^* Oversimplified model with two types of magnetically-coupled pairs RA…cation. *^e^* Calculations of exchange interactions between [CrTol_2_]^+^ and structurally disordered RAs. *^f^* *J* values calculated in the same pair at different mutual orientations of RAs.

## 5. Summary and Future Perspectives

Available synthetic methods allow preparation of various 1,2,5-chalcogenadiazoles (chalcogen: S, Se, Te) and their numerous functional derivatives, particularly areno- and hetareno-fused derivatives. Overall, molecular diversity is very broad in the field and can further be broadened by means of DFT calculations-aided molecular design.

Derivatives of the 1,2,5-chalcogenadiazole ring system possess positive EA which depends on molecular composition and structure and can be enlarged by electron-withdrawing substituents, *i.e.*, controlled. With various reducing agents, the compounds can be easily transformed into persistent RAs and the latter isolated in the form of thermally stable crystalline salts. The salts, both homospin (where only anions were paramagnetic) and heterospin (where both ions were paramagnetic) revealed mainly AF interactions in their spin systems. It should be noted that currently systems with AF interactions are receiving increased attention because of the experimental observation of the spin-liquid state, as well as their prospects in creating nanoscale memory cells [201,202].

With the McConnell I model dealing with spin polarization [187] dominance of AF interactions is expected for the homospin salts. In these salts, the spin density on the VdW surfaces of their RAs is mostly positive, with only small islands of negative spin density. For neighboring RAs in the crystal lattice, contacts of like spin density are most probable to give rise to AF exchange interactions between them, whereas for FM interactions, contacts of unlike spin density are required (except contacts of unlike density on orthogonal MOs leading to AF interactions). In general, the heterospin salts with paramagnetic sandwich cations possessing peripheral negative spin density on ligands are better suited for FM interactions and, therefore, are worth of further investigations.

Especially promising are heterospin salts with S = 1/2 cations [MAr_2_]^+^ (M = Cr, Mo, W). The IE of their precursors MAr_2_ (reducing agents in the target salts’ preparations) can be varied in a rather broad range depending on the ring substituents. With the same Ar ligands, the IE is practically equal for M = Cr (3d), Mo (4d), and W (5d) allowing one to cover the whole d block in a single approach. The cations with Mo and W are of special interest because of the strong spin-orbit coupling inherent in these heavy atoms. The strength of the spin-orbit coupling increases sharply with the atomic number as Z^4^ to be sufficient for atoms with Z > 30. In the heterospin salts containing Mo (Z = 42) or W (Z = 74) atoms in the cation and heavier chalcogen Se (Z = 34) or Te (Z = 52) atoms in the anion, the strong spin-orbit coupling can lead to spin canting to originate a FM ground state even under conditions of AF exchange interactions between paramagnetic centers (the Dzyaloshinsky−Moriya mechanism [187]). Successful experiments with MoAr_2_ reducing agents are already in progress.

Additionally to 1,2,5-chalcogenadiazoles, there are many other chalcogen-nitrogen π-heterocycles partially represented in Table 2, Table 3, Table 4, Table 5 and Table 6 and expected to be precursors of persistent RAs which can be isolated in the form of thermally stable salts, or effective electron acceptors in synthesis of new CT complexes. Therefore, one may hope that the discussed field of the chalcogen-nitrogen chemistry is very far from being exhausted one.
